# What effect does functional appliance treatment have on the temporomandibular joint? A systematic review with meta-analysis

**DOI:** 10.1186/s40510-019-0286-9

**Published:** 2019-08-12

**Authors:** Karma Shiba Kyburz, Theodore Eliades, Spyridon N. Papageorgiou

**Affiliations:** Clinic of Orthodontics and Pediatric Dentistry, Center of Dental Medicine, University of Zurich, Plattenstrasse 11, Zurich, Switzerland

**Keywords:** Orthodontics, Mandibular retrognathism, Functional appliance, Temporomandibular joint, Systematic review, Clinical trial

## Abstract

**Background:**

The aim of the current systematic review was to compare the radiologic effects of functional appliance Class II treatment compared to no treatment on the temporomandibular joint and its components.

**Methods:**

Nine databases were searched up to June 2019 for randomized or prospective non-randomized clinical trials comparing Class II patients treated with functional appliances to untreated patients. After duplicate study selection, data extraction, and risk of bias assessment with the Cochrane tool and the ROBINS-I tool, random effects meta-analyses of mean differences (MDs) and their 95% confidence intervals (CIs) were performed, followed by the assessment of the quality of evidence with GRADE.

**Results:**

A total of 11 papers on 8 unique trials with 377 patients (39.8% male; average age 10.3 years) were finally included. Limited evidence indicated that compared to untreated growing patients functional appliance treatment was associated with increased condylar width (2 studies; MD 1.1 mm; 95% CI 0.1 to 2.2 mm; very low evidence quality), decreased anterior joint space (2 studies; MD − 0.7 mm; 95% CI − 0.5 to − 0.9 mm; very low evidence quality), increased superior joint space (2 studies; MD 0.7 mm; 95% CI 0.5 to 1.0 mm; very low evidence quality), increased posterior joint space (2 studies; MD 1.0 mm; 95% CI 0.9 to 1.2 mm; very low evidence quality), and vertical displacement of the glenoid fossa (2 studies; MD 0.4 mm; 95% CI 0.1 to 0.7 mm; very low evidence quality). The main limitations affecting the validity of the present findings were the inclusion of non-randomized studies with methodological issues, imprecision due to limited samples of the included studies, and inconsistencies among studies.

**Conclusions:**

Currently existing evidence from controlled clinical studies on humans indicates that functional appliance treatment is associated with positional and skeletal alterations of the temporomandibular joint in the short term compared to untreated controls. However, the clinical relevance of these changes remains unclear, while the quality of existing evidence is low due to methodological issues of existing studies.

**Review registration:**

PROSPERO, CRD42018109271

**Electronic supplementary material:**

The online version of this article (10.1186/s40510-019-0286-9) contains supplementary material, which is available to authorized users.

## Introduction

Functional appliances are often employed for the treatment of Class II malocclusion associated with mandibular retrusion, which have historically attempted to stimulate mandibular growth [1, 2] and improve the facial profile [3].

Proof of concept for the skeletal effects of mandibular anterior repositioning with functional appliances was provided by animal studies [4, 5] and early clinical studies on humans [6–9] indicating mandibular length gains and induction of condylar growth. However, evidence from subsequent well-designed clinical trials and systematic reviews thereof [10–15] indicated that the actual sagittal position of the anterior border of the mandible is only slightly affected by functional appliance treatment and Class II occlusal relationship is mostly corrected by dentoalveolar effects.

Still, there are indications that mandibular anterior repositioning and the downward/forward displacement of the condyles induces an adaptive remodeling of the condyle and the glenoid fossa [16–18] and might modify the position of the articular disc [19, 20]. Clarifying the treatment-induced changes in the temporomandibular joint and its components is important in order to assess the stability of treatment-induced changes after mandibular advancement. Additionally, incidents of disc displacement after mandibular repositioning have been reported [21], even though others refute any deleterious effects on the temporomandibular joint [22].

However, the majority of existing clinical trials on this field have focused on occlusal or skeletal changes assessed through dental casts or lateral cephalograms. Robust assessments of the morphology of the skeletal/connective tissues of the temporomandibular joint necessitate imaging techniques with increased discerning ability for the joint region like computed tomography (CT), cone beam computed tomography (CBCT), or magnetic resonance imaging (MRI) [23–26]. A previous systematic review from 2015 assessed the effect of fixed mandibular repositioning devices on TMJ morphology [27]. However, its search covered only studies published to mid-2015, included only fixed appliances, did not factor out normal growth of the TMJ by including untreated controls, did not use the novel tool from the Cochrane Collaboration to assess the risk of bias of included non-randomized studies [28], could not perform any meta-analyses, and did not assess the quality of clinical recommendations that can be drawn from existing evidence. Therefore, it was judged that a new systematic review needed to be conducted.

The aim of the present systematic review of clinical studies was to assess the effect of functional appliance treatment on the temporomandibular joint morphology of patients with Class II malocclusion compared to untreated patients.

## Material and methods

### Protocol, eligibility criteria, and registration

This review’s protocol was a priori registered in PROSPERO (CRD42018109271), its literature searches transparently reported (Additional file 1: Appendix 1), and all post hoc changes were appropriately noted (Additional file 1: Appendix 2). This systematic review was conducted and reported according to the Cochrane Handbook [28] and PRISMA statement [29], respectively.

Based on the Participants-Interventions-Comparisons-Outcome-Study design (PICOS) approach, we included randomized clinical trials or non-randomized controlled clinical trials on human adolescent patients of any age or sex with Class II malocclusion treated with removable or fixed functional appliance and skeletal condylar growth as the primary outcome. Secondary outcomes included joint space, condyle-fossa relationship, condyle-disc relationship, condyle-disc-fossa relationship, skeletal mandibular growth, disc formation, and disc position. Animal studies, in vitro studies, and studies of patients with obstructive sleep apnea, juvenile idiopathic arthritis, rheumatoid arthritis, psoriasis, syndromes, fractures, surgical intervention, Class III, or osteoporosis were excluded.

### Information sources and literature search

The following nine electronic databases were systematically searched for this review: MEDLINE (via PubMed), Embase, The Cochrane Library (CDSR, CENTRAL, and DARE), Virtual Health Library (including Bibliography Brazilian Dentistry and LILACS), Scopus, ISI Web of Knowledge, and ClinicalTrials.gov (Additional file 1: Appendix 1). Manual search was applied on the databases Directory of Open Access Journals (DOAJ), Digital Dissertations (via UMI Proquest), metaRegister of Controlled Trials, WHO trials search portal, and Google Scholar for additional trials as well as for the reference lists of the included studies. The search was made without any limitations from inception of each database up to June 16, 2019. Aside from filtering trials on humans, no other filters for language, publications year, and status were applied.

### Study selection and data collection

The identified studies from the literature search were sequentially screened by title, abstract, and full text by one author (KSK) with subsequent duplicate independent checking against the eligibility criteria by another author (SNP), while conflicts were resolved by a third author (TE).

The same protocol was applied for the extraction of study characteristics (study design, setting, country, patient number, sex, age, appliances, treatment duration, timing of follow-up, and outcome measured) and for the numerical data collection using pre-defined forms. Piloting of the forms was performed during the protocol stage until over 90% agreement was reached, when any data was missing in the trial, if possible.

### Risk of bias in individual studies

The risk of bias was evaluated using the Cochrane risk of bias tool [28] for randomized trials and the Risk Of Bias In Non-randomized Studies-of Interventions (ROBINS-I) tool [30] for non-randomized studies. This assessment was performed by one author (KSK) and independently checked by another author (SNP).

### Data synthesis

The primary outcome of this systematic review was the change in the linear/volumetric joint space, measured as the distance/volume between the functional surface of the condyle and the articular eminence. Secondary outcomes included the anterior/posterior angle between the anterior/posterior disc band and the condylar line, the condylar coronary width, the displacement of the glenoid fossa, and the condyle’s sagittal concentricity. All additional outcomes reported in included studies are also listed, but only briefly analyzed.

Data was summarized and considered suitable for pooling if similar intervention and/or control groups were compared and if similar outcomes were reported. All existing studies were included in the analysis independently of reporting completeness, if possible; where data was missing, they were calculated from existing data or requested them from the authors. For studies reporting on data before and after treatment, but not on the treatment-induced changes, we calculated those with a moderate pre-post correlation of 0.75. Mean differences (MDs) of treatment changes for continuous outcomes and relative risks (RRs) for binary outcomes and their corresponding 95% confidence intervals (CIs) were calculated. As the effects of functional appliance treatment were deemed to be highly variable according to patient age, sex, individual maturation of the maxillofacial structures, and appliance characteristics [14, 15], a random-effects model was chosen to calculate the average distribution of treatment effects that can be expected [31]. A restricted maximum likelihood random-effects variance estimator was used instead of the older DerSimonian-Laird one, following recent guidance [32]. Random effects 95% predictions were to be calculated for meta-analyses with at least three studies to aid in their interpretation by quantifying expected treatment effects in a future clinical setting [33].

The extent and impact of between-study heterogeneity were assessed by inspecting the forest plots and by calculating the tau-squared and the *I*-squared statistics, respectively. The 95% CIs (uncertainty intervals) around tau-squared and the *I*-squared were calculated to judge our confidence about these metrics. We arbitrarily adopted the *I*-squared thresholds of > 75% to be considered as signs of considerable heterogeneity, but we also judged the evidence for this heterogeneity (through the uncertainty intervals) and the localization on the forest plot.

A two-tailed *P* value of 0.05 was considered significant for all hypothesis testing, except for a 0.10 used for the test of heterogeneity and reporting biases. All analyses were run in Stata SE 14.0 (StataCorp, College Station, TX) by one author (SNP), and the study’s dataset was openly provided [34].

### Risk of bias across studies and additional analyses

Subgroup analyses, meta-regressions, assessments of reporting biases, and sensitivity analyses were initially planned in the review’s protocol but could ultimately not be conducted due to a limited number of included trials (Additional file 1: Appendix 2).

The overall quality of clinical recommendations (confidence in effects estimates) for each of the main outcomes was rated using the Grades of Recommendation, Assessment, Development, and Evaluation (GRADE) approach [35] and an improved Summary of Findings table format [36]. The optimal information size was estimated for each outcome independently to be able to identify a minimal clinical important effect with an average standard deviation (based on this review’s study sample), with type I and type II errors set at 5% and 20%, respectively. The minimal clinical important, large, and very large effects were conventionally defined as half, one, and two standard deviations for continuous outcomes and as relative risks of 1.5, 2.5, or 5.0 for binary outcomes [37]. This assessment of the risk of bias for among-trials was conducted independently by two authors (SNP and KSK), and discrepancies were resolved by a third author (TE).

## Results

### Study selection

**Fig. 1:**
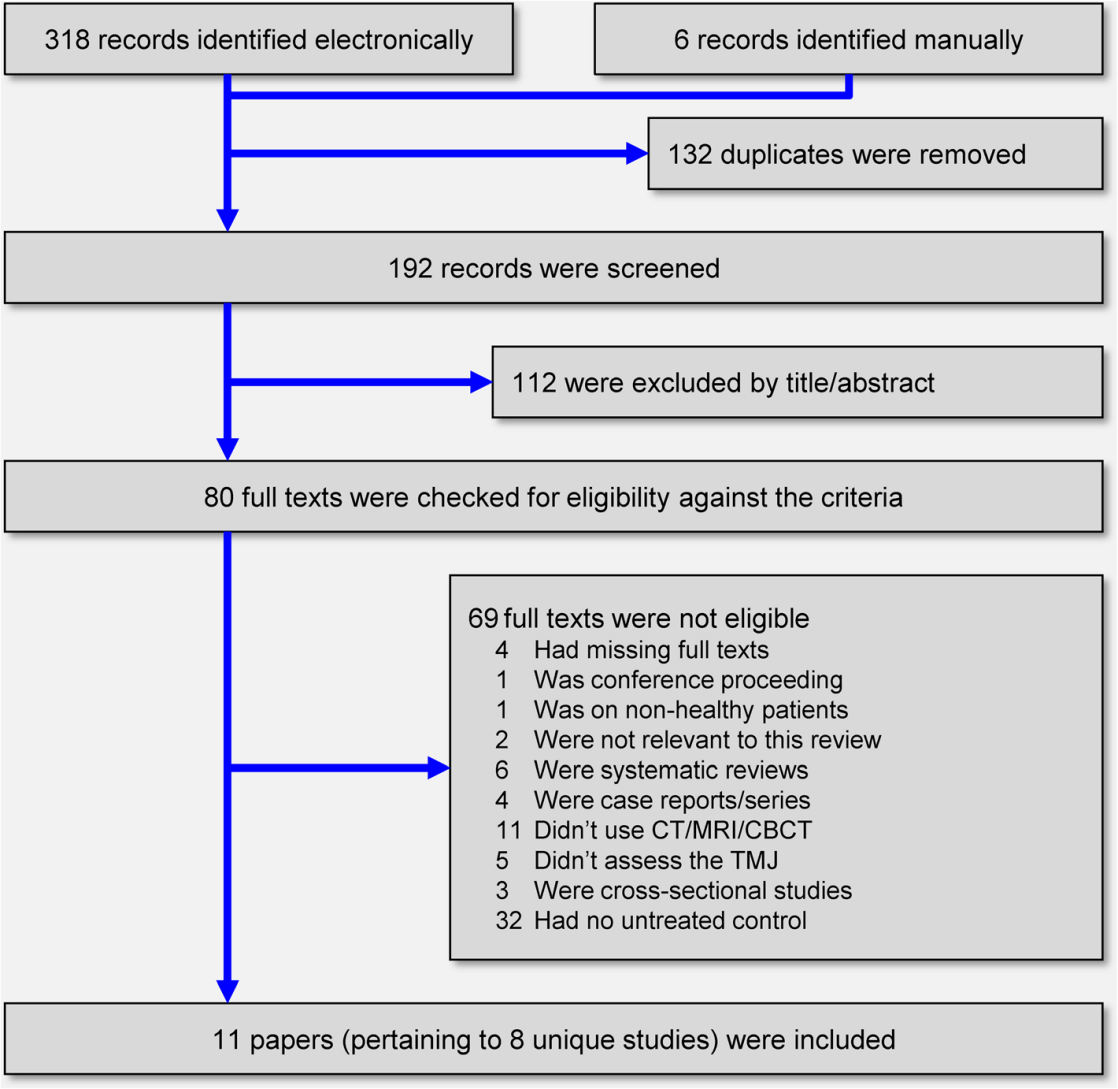
PRISMA flow diagram for identification and selection of eligible trials

The electronic literature yielded a total of 318 records, while 6 more were identified manually (Fig. 1). After the removal of duplicates and screening of titles and abstracts, 80 full-text papers were scrutinized against the eligibility criteria. After applying these eligibility criteria, a total of 11 publications pertaining to 8 unique clinical studies were finally included in this systematic review (Additional file 1: Appendix 3).

### Study characteristics

Three randomized clinical trials and 5 non-randomized comparative cohort studies were finally included, the characteristics of which can be seen in Table 1. The included studies were conducted in private practices or university clinics in six different countries (Brazil, Egypt, India, Thailand, Turkey, the USA) and had been published as journal papers and/or dissertations in English or Portuguese between 1999 and 2018. A wide variety of removable or fixed functional appliances were used that include Activator, Bionator, Forsus Nitinol Flat-Spring, Fränkel, Herbst (combined with maxillary expansion), and Twin Block. All control groups were concurrently recruited, except from a single study that employed a historical control group from a longitudinal growth study. These 6 trials included a total of 377 patients treated with functional appliances or observed with an average sample size of 47 patients/study (range 18–80). Among the 7 studies reporting gender, 129/324 (39.8%) were male, while 1 study included only female patients. Among the 6 studies reporting age, the average patient age was 10.3 (mean ages within each study ranging between 8.5 and 11.7 years). Treatment outcome in the included studies was measurement with MRI, CT, or CBCT before and after treatment, to follow-up periods ranging from 6.0 to 18.0 months. Assessed outcome included joint space (distance or volume), condyle position or volume, disc position or concentricity, glenoid fossa position or volume, and skeletal morphology (assessed with geometric morphometrics).

**Table 1:** Characteristics of included studies

Study	Design; setting; country^$^	Patients (M/F); age*	Intervention; duration^#^	Follow-up	Imaging method	Outcome
Arat et al. [38]	uNRS; Uni; TUR	CI. II/1; ANB ≥ 4°; SN-ML 25-32° EG, 9 (2/7); 11.2 CG, 9 (4/5); 9.7	Activator; 16.0	Pre-Tx 6.0 mos post-Tx (24.0 mos)	MRI	Condyle-to-disc angle (ant/mid/post) Joint space (anterior/posterior/medial)
Arici et al. [39]	RCT; Uni; TUR	CI. II/1; OJ > 5 mm; Mnd RTG EG, 30 (13/17); NR CG, 30 (9/21); NR	FNFS; 7.0	Pre-Tx Post-Tx (7.0 mos)	CT	COND volume GF volume Joint space volume (anterior/posterior)
Cevidanes et al. [40, 41]	RCT; Uni; USA	CI. II/1 ≥ ¾ unit; OJ > 4.5-10.0 mm EG, 28 (NR); 10.3 CG, 25 (NR); 10.9	Fränkel-2; 18.0	Pre-Tx Post-Tx (18.0 mos)	MRI	PCA of skeletal morphology
Chavan et al. [42]	uNRS; Uni; IND	CI. II/1 EG1, 10 (6/4); 12.5 EG2, 10 (4/6); 11.5 CG, 10 (3/7); 12.0	EG1: Twin Block EG2: Bionator	Pre-Tx 6.0 mos in Tx	MRI	SAG disc concentricity SAG disc position
Chintakanon and Chintakanon et al. [43, 44]	pNRS; pract; THA	CI. II/1; OJ > 5 mm; Mnd RTG EG, 19 (14/5); 11.7 CG, 21 (13/8); 11.5	Twin Block; 6.0	Pre-Tx 6.0 mos in Tx	MRI	Condylar axial angle Coronal disc position Eminence angle SAG disc concentricity SAG disc position
Croft et al. [45]	rNRS; pract; USA	ANB ≥4°; Cl. II ≥ ½ unit EG, 40 (16/24); 8.5 CG^†^, 40; NR (matched)	RME/Herbst/EGA; 11.0	Pre-Tx Post-Tx (11.0 mos) 2.7 years post-Tx	CT	Condylar growth GF displacement Joint space (anterior/posterior/superior)
Elfeky et al. [46]	uNRS; Uni; EGY	Cl. II ≥ ½ unit; Mnd RTG; V-pattern EG, 22 (0/22); NR CG^†^, 18 (0/18); NR	Twin Block; 9.4	Pre-Tx Post-Tx (9.4 mos)	CBCT	COND position COND size GF position Joint space (anterior/posterior/superior/medial)
Franco et al. and Franco [47, 48]	RCT; Uni; BRA	CI. II/1 EG, 28 (15/13); 10.3 CG, 28 (14/14); 10.9	Fränkel-2; 18.0	Pre-Tx Post-Tx (18.0 mos)	MRI	Disc position Disc shape

*CG* control group, *COND* condyle, *EG* experimental group, *EGA* eruption guidance appliance, *FA* functional appliance (unspecified), *FNFS* Forsus nitinol flat-spring, *GF* glenoid fossa, *M* male, *RCT* randomized clinical trial, *Mnd RTG* mandibular retrognathism, *NR* not reported, *PCA* principal component analysis, *pNRS* prospective non-randomized study, *Pract* practice, *rNRS* retrospective non-randomized study, *SAG* sagittal, *Uni* university, *V* vertical, *mos* months

^#^Duration of active Class II treatment in months

^$^Countries are given with their ISO-3 code

*Age is given in years either as mean

^†^Historical

### Risk of bias within studies

**Fig. 2:**
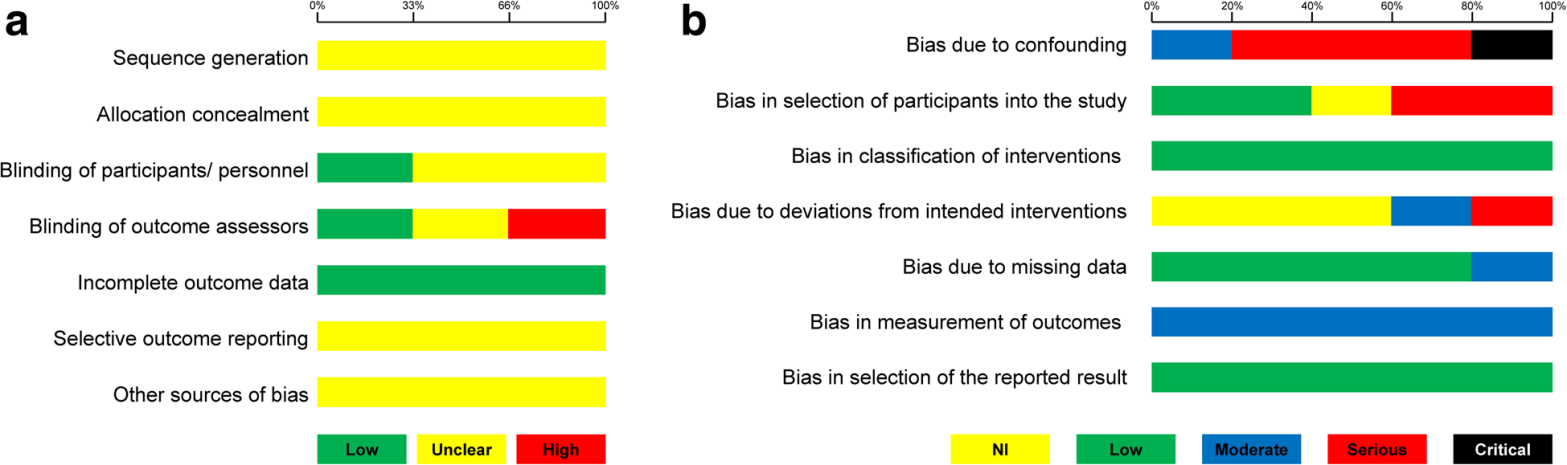
**a** Risk of bias summary for included randomized trials with the Cochrane risk of bias tool. **b** Risk of bias summary for included non-randomized trials with the ROBIN-I tool

The risk of bias of included randomized trials was high for one trial (due to detection bias) and unclear for the remaining two. It is important here to note that the vast majority of bias domains for the three included trials could not be adequately assessed, due to their poor reporting quality (Table 2; Fig. 2a). The risk of bias of all included non-randomized trials according to the ROBINS-I tool was found to be serious or critical (Table 3; Fig. 2b). The most problematic issues identified pertained to confounding, selection bias, performance bias, and detection bias.

**Table 2:** Risk of bias of the included randomized clinical trials with the Cochrane risk of bias tool

Trial	Sequence generation	Allocation concealment	Blinding of participants/personnel	Blinding of outcome assessors	Incomplete outcome data	Selective outcome reporting	Other sources of bias
Arici et al. [39]	Unclear—no randomization details provided information provided: “Thirty patients (17 girls, 13 boys) were randomly assigned to treatment with a fixed functional orthodontic appliance (Forsus nitinol flat-spring) for 6 to 9 months (mean, 7 months).”	Unclear—no information provided.	Unclear—blinding is impractical for both patients and clinician; outcome is objective, but was not assessed blindly.	High risk—no mention of blinding throughout the paper; blinding could have been implemented.	Low risk—no drop-outs or patient losses are reported.	Unclear—it is difficult to judge whether selective reporting is a problem, as no protocol exists.	Unclear—no definite issue identified (except for a possible confounder of vertical skeletal configuration type).
Cevidanes et al. [40, 41]	Unclear—no randomization details provided: “The Class II subjects were randomly allocated to 2 subgroups, treated and control, to avoid bias in the group comparison.”	Unclear—no information provided.	Low risk—blinding is impractical for both patients and clinician; outcome is objective and was assessed blindly.	Low risk—all images were coded and their order permutated to keep the analyst blind to subject identification group, and timing (T1 or T2).	Low risk—no drop-outs or patient losses are reported.	Unclear—it is difficult to judge whether selective reporting is a problem, as no protocol exists.	Unclear—no definite issue identified (except for a possible confounder of vertical skeletal configuration type).
Franco et al. and Franco [47, 48]	Unclear—no randomization details provided: “The sample was randomly dichotomized into 2 subgroups, treated subjects and untreated controls, to avoid bias in the group comparison.”	Unclear—no information provided.	Low risk—blinding is impractical for both patients and clinician; outcome is objective and was assessed blindly.	Unclear—blinding is mentioned: “A double-blind procedure was used”. However, no details are given and this is not mentioned at all in study published subsequently as dissertation.	Low risk—no drop-outs or patient losses are reported.	Unclear—it is difficult to judge whether selective reporting is a problem, as no protocol exists.	Unclear—no definite issue identified (except for a possible confounder of vertical skeletal configuration type).

**Table 3:** Risk of bias of the included non-randomized clinical trials with the ROBINS-I tool

	Bias due to confounding	Bias in selection of participants into the study	Bias in classification of interventions	Bias due to deviations from intended interventions	Bias due to missing data	Bias in measurement of outcomes	Bias in selection of the reported result	Overall bias
Arat et al. [38]	Serious	No information	Low	No information	Low	Moderate	Low	Serious
Chavan et al. [42]	Serious	Low	Low	No information	Low	Moderate	Low	Serious
Chintakanon and Chintakanon et al. [43, 44]	Serious	Low	Low	Moderate	Low	Moderate	Low	Serious
Croft 1999 [45]	Critical	Serious	Low	Serious	Low	Moderate	Low	Critical
Elfeky et al. [46]	Moderate	Serious	Low	No information	Moderate	Moderate	Low	Serious

### Results of individual studies and synthesis of results

The results of all extracted outcomes from each included study trial are given in Additional file 1: Appendix 4, filtered naively by statistical significance (at 5%) and clinically relevance (judged as having an effect at least equal to one deviation of the control group’s response). Clinically relevant changes from functional appliance treatment were identified at the joint space, where shrinking of the anterior and widening of the posterior/superior joint space was seen. Insertion of the functional appliances leads to an anterior position of the condyle that was followed by a repositioning of the condyles back in their glenoid fossa after treatment. Although the condyles appeared to be seated in their fossae, the position of the condyle relative to the fossa was still anterior to its pretreatment position, while the disc was also moved more anteriorly compared to the control group. Additionally, the results of a morphometrics study [40, 41] indicated that the condyle exhibited increased vertical displacement/remodeling components compared to untreated Class II controls, which was on the opposite direction of the gonion displacement/remodeling.

**Fig. 3:**
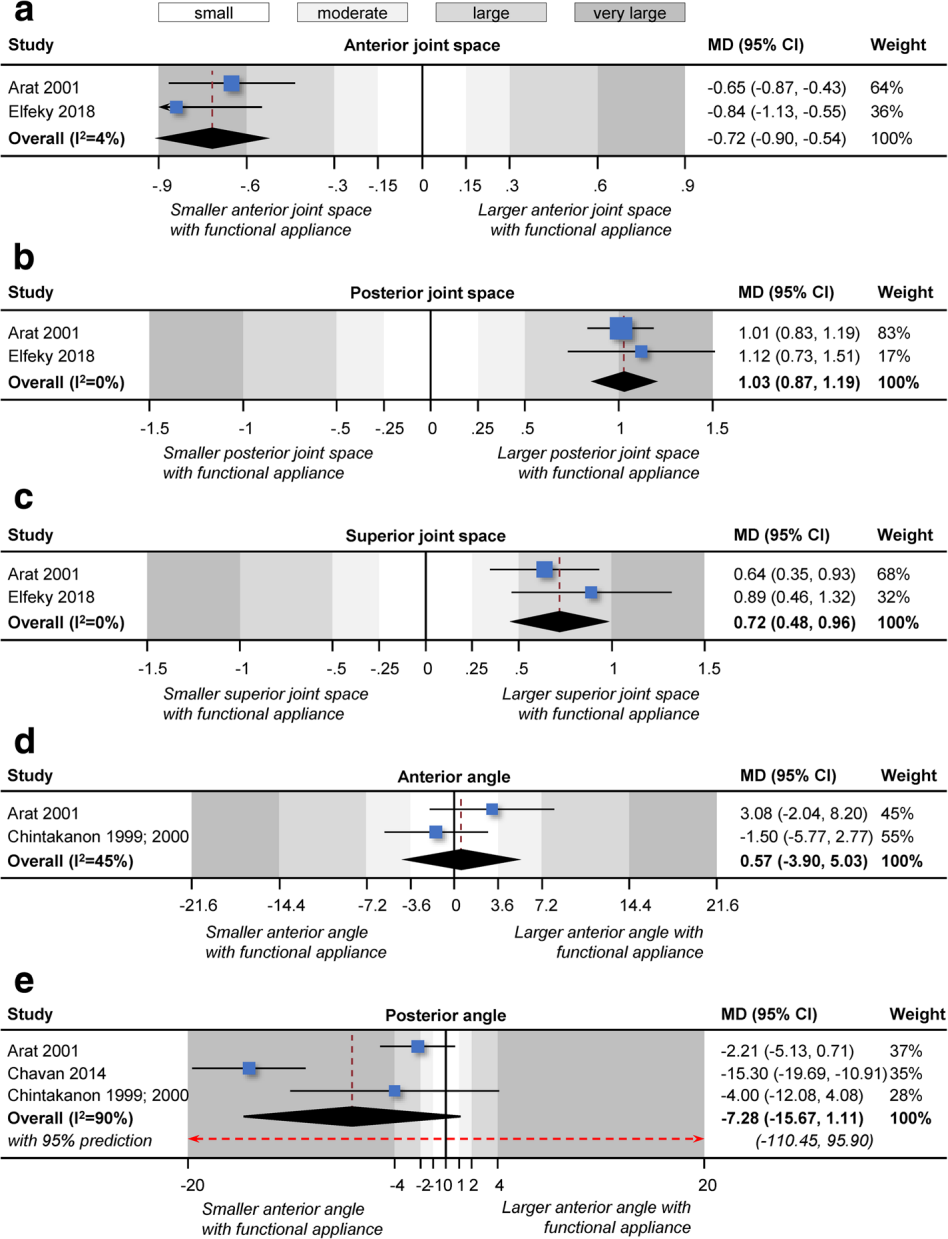
Contour-enhanced forest plots for random effects meta-analyses comparing functional appliance treatment to no treatment (observation) in terms of changes in **a** anterior joint space, **b** posterior joint space, **c** superior joint space, **d** anterior angle, and **e** posterior angle

**Fig. 4:**
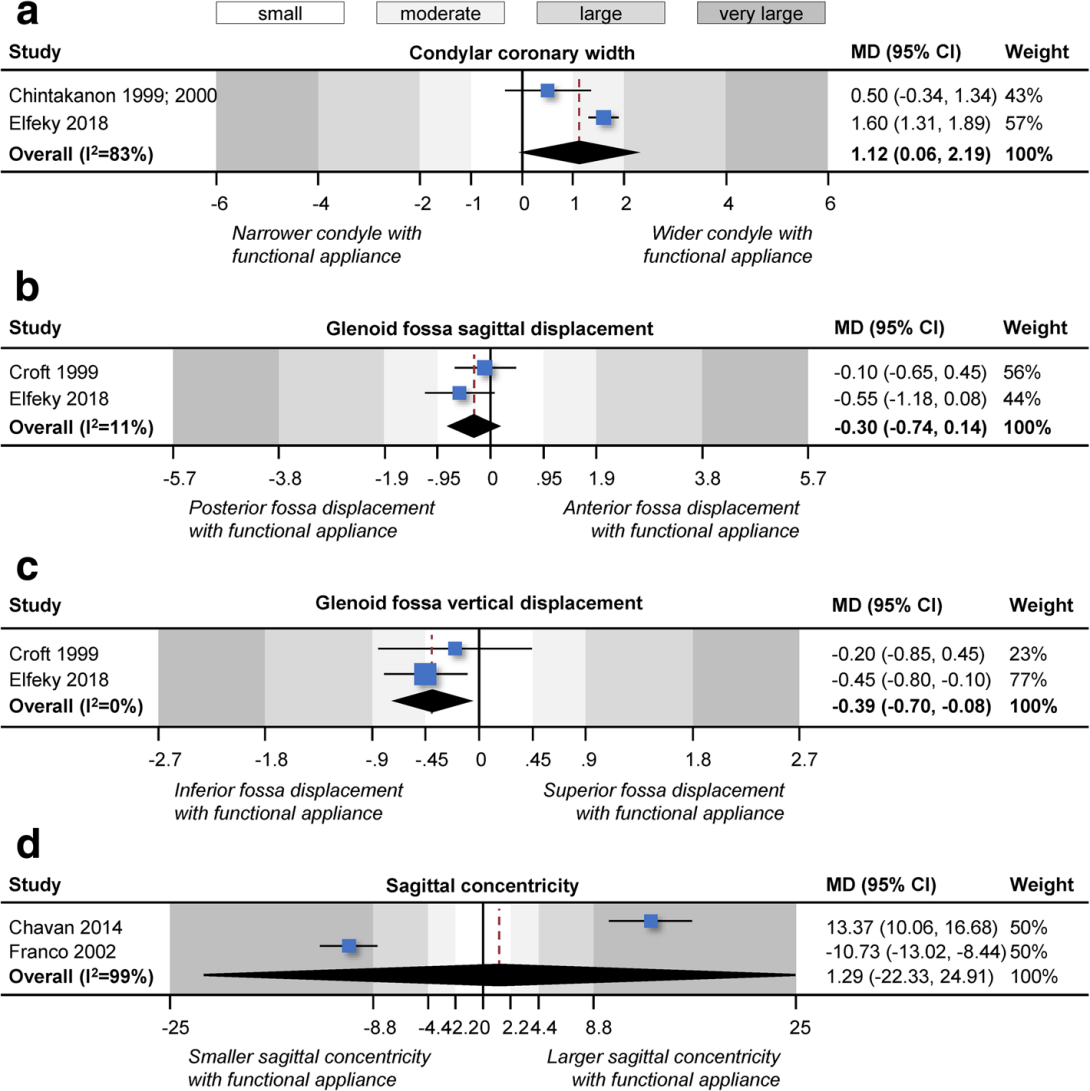
Contour-enhanced forest plots for random effects meta-analyses comparing functional appliance treatment to no treatment (observation) in terms of changes in **a** condylar coronary width, **b** glenoid fossa sagittal displacement, **c** glenoid fossa vertical displacement, and **d** sagittal concentricity. CI, confidence interval; MD, mean difference

Quantitative pooling (meta-analyses) of at least two studies could be performed for nine outcomes: anterior joint space, posterior joint space, superior joint space, anterior angle, posterior angle, condylar coronary width, glenoid fossa sagittal displacement, glenoid fossa vertical displacement, and sagittal concentricity (Table 4; Figs. 3 and 4). Statistically significant and clinically relevant changes in the joint space were seen after 6.0–11.0 months of functional appliance treatment, which were translated to statistically large to very large reduction in anterior joint space (2 studies; MD = − 0.7 mm; 95% CI = − 0.9 to − 0.5 mm), increase in posterior joint space (2 studies; MD = 1.0 mm; 95% CI = 0.9 to 1.2 mm), and increase in superior joint space (2 studies; MD = 0.7 mm; 95% CI = 0.5 to 1.0 mm). These effects were fairly consistent and homogenous between studies (*I*^2^ = 0–4%). This was accompanied by a similarly consistent vertical displacement of the glenoid fossa (MD = − 0.4 mm; 95% CI = − 0.7 to − 0.1 mm; *I*^2^ = 0%), which was however of small to moderate magnitude. Finally, a statistically significant increase in condylar coronary width compared to untreated controls was seen (2 studies; MD = 1.1 mm; 95% CI = 0.1 to 2.2 mm), which was however of small to moderate magnitude and more heterogeneous (*I*^2^ = 83%).

**Table 4:** Results of random effects meta-analyses performed

	Treatment effects			Heterogeneity		
Outcome	*n*	MD (95% CI)	*P*	tau^2^ (95% CI)	*I*^2^ (95% CI)	95% prediction
Anterior joint space	2	− 0.72 (− 0.90, − 0.54)	< 0.001	0 (0, 2.25)	4% (0%, 99%)	NC
Posterior joint space	2	1.03 (0.87, 1.19)	< 0.001	0 (0, 1.41)	0% (0%, 98%)	NC
Superior joint space	2	0.72 (0.48, 0.96)	< 0.001	0 (0, 3.92)	0% (0%, 99%)	NC
Anterior angle	2	0.57 (− 3.90, 5.03)	0.80	4.71 (NC)	45% (NC)	NC
Posterior angle	3	− 7.28 (− 16.67, 1.11)	0.09	47.61 (7.23, 921.80)	90% (7%, 99%)	− 110.45, 95.90
Condylar coronary width	2	1.12 (0.06 2.19)	0.04	0.50 (0, 75.91)	83% (0%, 100%)	NC
Glenoid fossa sagittal displacement	2	− 0.30 (− 0.74, 0.14)	0.18	0.01 (0, 12.63)	11% (0%, 99%)	NC
Glenoid fossa vertical displacement	2	− 0.39 (− 0.70, − 0.08)	0.01	0 (0, 5.027)	0% (0%, 99%)	NC
Sagittal concentricity	2	1.29 (− 22.33, 24.91)	0.92	288.29 (NC)	99% (NC)	NC

*CI* confidence interval, *MD* mean difference, *NC* noncalculable

### Risk of bias across studies, additional analyses, and quality of evidence

No formal assessment of risk of bias across studies or any subgroup/sensitivity analyses could be performed due to the limited number of included trials in the meta-analyses, which would be rendered instable by trial omissions.

The quality of evidence for all performed meta-analyses was very low according to the GRADE approach, due to the inclusion of non-randomized studies, the methodological inadequacies of included studies, imprecision of the estimated effects, and inconsistency across studies (Table 5; Additional file 1: Appendix 5). Therefore, our confidence in the observed alterations in the TMJ associated with functional appliance treatment is hampered and future studies might change current recommendations.

**Table 5:** Summary of findings table according to the GRADE approach

	Anticipated absolute effects (95% CI)				
Outcome (follow-up) Studies (patients)	Controla	FA	Difference with FA	Quality of the evidence (GRADE)b	What happens with FAs
Anterior joint space (6.0–9.4 mos) 54 patients (2 studies)	< 0.1 mm	–	0.7 mm smaller (0.5 to 0.9 smaller)	⊕_◯⃝⃝_ very low^c^ Due to bias	Might shrink anterior joint space
Posterior joint space (6.0–9.4 mos) 54 patients (2 studies)	− 0.1 mm	–	1.0 mm larger (0.9 to 1.2 larger)	⊕_◯⃝⃝_ very low^c^ Due to bias	Might enlarge posterior joint space
Superior joint space (6.0–9.4 mos) 54 patients (2 studies)	− 0.2 mm	–	0.7 mm larger (0.5 to 1.0 larger)	⊕_◯⃝⃝_ very lowc Due to bias	Might enlarge superior joint space
Anterior angle (6.0 mos) 58 patients (2 studies)	− 0.8°	–	0.6° larger (3.9 smaller to 5.0 larger)	⊕_◯⃝⃝_ very lowc^, d^ Due to bias, imprecision	Little to no difference in anterior angle
Posterior angle (6.0 mos) 88 patients (3 studies)	− 1.4°	–	7.3° smaller (16.7 smaller to 1.1 larger)	⊕_◯⃝⃝_ very lowc^, d^ Due to bias, imprecision	Little to no difference in posterior angle
Condylar coronary width (6.0–9.4 mos) 76 patients (2 studies)	− 0.1 mm	–	1.1 mm wider (0.1 to 2.2 wider)	⊕_◯⃝⃝_ very lowc^, d^ Due to bias, imprecision	Might increase condylar coronary width
GleFo sagittal displacement (9.4–11.0 mos) 164 patients (2 studies)	− 0.9 mm (posterior)	–	0.3 mm more posterior (0.7 less to 0.1 more)	⊕_◯⃝⃝_ very low^c^ Due to bias	Little to no difference in glenoid fossa sagittal displacement
GleFo vertical displacement (9.4–11.0 mos) 164 patients (2 studies)	0.7 mm (superior)	–	0.4 mm more inferior (0.7 to 0.1 more)	⊕_◯⃝⃝_ very low^c^ Due to bias	Little to no difference in glenoid fossa vertical displacement
Sagittal concentricity index (6.0–18.0 mos) 86 patients (2 studies)	1.3%	–	1.3% greater (22.3 smaller to 24.9 greater)	⊕_◯⃝⃝_ very low^c, d, e^ Due to bias, imprecision, inconsistency	Little to no difference in sagittal concentricity

Intervention: functional appliance treatment (Activator, Bionator, Forsus Nitinol Flat-Spring, Fränkel, Herbst, Twin Block) versus control (observation)/population: Class II adolescent patients/setting: university clinics, private practice (Brazil, Egypt, India, Thailand, Turkey, USA)

*CI* confidence interval, *FA* functional appliance, *GleFo* glenoid fossa, *GRADE* Grading of Recommendations Assessment, Development and Evaluation, *mos* months

^a^Response in the control group is based on average response of included studies

^b^Starts from “low,” due to the inclusion of randomized studies

^c^Downgraded by one point for risk of bias (serious/critical risk of bias due to methodological limitations)

^d^Downgraded by one point due to imprecision, as the optimal information size was judged not to be met and/or the summary estimate was strewn across different categories of effect magnitude

^e^Downgraded one for inconsistency (*I*^2^ > 75%), which can affect our decision about the treatment effects

## Discussion

### Summary of findings

This systematic review summarizes evidence from clinical studies on the effect of functional appliance Class II treatment on the temporomandibular joint. Even though functional appliances have been used for many decades to treat Class II malocclusion, only 8 small clinical controlled studies with 377 were identified and found eligible for inclusion in this review.

As far as skeletal changes of the condyle or the glenoid fossa are concerned, some evidence indicated that patients treated with functional appliances differed from untreated patients. Meta-analysis of two studies indicated that the condyles of treated patients presented increased coronary width 6 to 9 months post-treatment compared to untreated controls (MD = 1.1 mm; Table 4). However, the effect was of small to moderate magnitude and close to the measurement error (Fig. 3b), while the quality of evidence was very low due to bias and imprecision (Table 5). Similar findings were observed by two included studies [39, 46] that reported a small increase in condylar dimensions and volume 7 to 9 months after functional appliance treatment compared to untreated controls (Additional file 1: Appendix 4). Interestingly, the same study found no significant increase in the volume of the glenoid fossa could be found (Additional file 1: Appendix 4). Finally, included studies indicated that functional appliance treatment was associated with increased posterior growth of the condyles [45] and increased vertical growth of the rami [40, 41] compared to untreated patients. Increased condylar growth activity after Class II treatment with functional appliances has also been confirmed in a study using single-photon emission CT [16], even though condylar growth activity was assessed only among treated patients and only in the short term.

However, positional differences for the various components of the temporomandibular joint were associated with functional appliance treatment. Meta-analysis of two studies indicated that the glenoid fossa of treated patients had been displaced more inferior 9 to 11 months post-treatment compared to untreated patients (MD = 0.4 mm; Table 4), but this effect was small (Fig. 3b) and supported by very low quality of evidence (Table 5). Additionally, the position of the condyle within the temporomandibular joint was also altered through functional appliance treatment. Meta-analysis of two studies indicated that 6–9 months post-treatment, the temporomandibular joints of treated patients presented shrunken anterior joint space (MD = 0.7 mm), enlarged posterior joint space (MD = 1.0 mm), and enlarged superior joint space compared to untreated patients (MD = 0.7; Table 4). This translates to a statistically large to very large forward and downward movement of the condyle within the temporomandibular joint (Fig. 3a), for which the quality of evidence was very low. Even though the magnitude of these effects is statistically speaking large to very large (larger than two standard deviations of the control group), their clinical relevance is debatable. This was confirmed from another two included studies: one [39] that measured anterior/posterior/superior joint space volume and one [46] that reported significant sagittal displacement of the condyle (MD = 1.3 mm; Additional file 1: Appendix 4). However, all included studies followed their patients only for a limited period ranging between 6 and 9 months (Table 5). It has been reported that although an anterior repositioning of the condyle relative to the glenoid fossa is seen in the short term after functional appliance treatment with Herbst, 1 year afterwards, the condyle is restored to its normal position in the glenoid fossa [22, 45]—presumably due to increased remodeling [44]. Other researchers however suggest that the use of semi-rigid functional appliances like the Forsus appliance might be preferable to rigid functional appliances like the Herbst or MARA appliance, since the former might enable better condylar repositioning post-treatment [39].

Interestingly, no consistent and significant change in the anterior or posterior angle was seen, which means that the relationship of the condylar disc to the condyle was not necessarily altered during functional appliance treatment. This was confirmed by the results of three included studies that found that functional appliance treatment did not significantly affect disc position [44], disc shape [47], or disc displacement [47]. This is in agreement with another cohort study of Class II patients treated with Herbst [22], which indicated that functional appliance treatment had a very minor positive effect, if any, on the condylar disc position. It might also be important here to note that measurement of the condylar position like the anterior and the posterior angle use as reference points the anterior and posterior limits of the disc, which are often difficult to identify on MRIs [23]. Additionally, changes in the anterior/posterior limit of the disc might not necessarily correspond to positional changes of the disc, but rather act as triggering mechanisms for adaptive activity during repositioning of the condyle, due to their anatomical connections to adjacent structures [38, 49].

As far as the performance of the used imaging modalities is concerned, MRI has been shown to be an accurate method for the assessment of soft and hard tissues of the TMJ with 95% and 93% accuracy for the identification of the disc position and osseous changes, respectively [26]. Likewise, conventional CT has been found to be equally accurate in imaging of the TMJ area in terms of disc displacement identification [50]. However, other researchers report that CBCT with a voxel size of 0.125 mm is considerably more accurate in identifying osseous changes of the TMJ than MRI [51]. Compared to CBCT, MRI seems to possess low sensitivity, but good specificity [51]. However, it might be prudent for patients with a diagnosed disc pathology or deformity to also examine them with CBCT to more precisely identify any changes of the hard tissues [52]. In the present review, included studies mostly used MRI to appropriately assess disc relationships and CT or CBCT to assess the bony structures, even though the included CBCT study [46] did not report the used voxel size.

### Strengths and limitations

The strengths of this systematic review consist of the registration of its a priori protocol in PROSPERO [53, 54], its exhaustive literature search, its improved analytical methods [32], the use of the GRADE approach [35] to assess the quality of the meta-evidence, and the transparent provision of the study’s data [34, 55].

However, certain limitations also exist. First and foremost, due to the limited studies on the field, also non-randomized studies were included that are more prone to bias than randomized trials [53], had several methodological limitations [56–58], and one study included a historical control group that might introduce further bias [59]. Furthermore, the identified studies were predominantly small and this might introduce small-study effects [60]. Additionally, the observed effects were of relatively small magnitude and might not necessarily translate to clinically relevant functional TMJ differences, which was not within the scope of the present review. Finally, the limited number of included studies and their suboptimal reporting did not enable assessments of heterogeneity, as well as the conduct of several analyses for different subgroups, small-study effects, and reporting biases that were planned to assess the robustness of the analyses [61].

## Conclusions

Currently existing evidence from controlled clinical studies on humans indicates that functional appliance treatment is associated with positional and skeletal alterations of the temporomandibular joint in the short term compared to untreated controls. These are mostly summarized by an anterior and inferior repositioning of the condyle, vertical displacement of the glenoid fossa, and increased condylar growth. However, the clinical relevance of these changes remains unclear, while the quality of existing evidence is low due to methodological issues of existing studies.

## Additional file

Additional file 1: Contains Appendices 1–5. (PDF 224 kb)

## Data Availability

All data generated or analyzed during this study are included in this published article or its supplements, while its dataset is openly provided through Zenodo (10.5281/zenodo.2648145).

## Abbreviations

CBCT: Cone beam computed tomography
CI: Confidence interval
CT: Computed tomography
GRADE: Grading of Recommendations Assessment, Development and Evaluation
MD: Mean difference
MRI: Magnetic resonance imaging
PICOS: Participants-Interventions-Comparisons-Outcome-Study design
RCT: Randomized clinical trial
REML: Restricted maximum likelihood
RR: Relative risk
TMJ: Temporomandibular joint
