# Supplementary material for: What effect does functional appliance treatment have on the temporomandibular joint? A systematic review with meta-analysis
Source: Prog Orthod. 2019 Aug 12;20:32. doi: 10.1186/s40510-019-0286-9 (PMC6689567; doi:10.1186/s40510-019-0286-9)
Supplement: Supplementary file 1 — Contains Appendices 1–5. (PDF 224 kb) [file 40510_2019_286_MOESM1_ESM.pdf]

**Supplementary material**
**Appendix 1.** Literature searched conducted to identify eligible studies (last search date June 16th, 2019).

| Database | Search | Limits | Hits |
|---|---|---|---|
| MEDLINE (via Pubmed) | (orthodon* OR malocclusion OR orthopedics OR "Class II" OR prognath* OR retrognath*) AND ("functional appliance" OR "functional treatment" OR Activator OR Andresen OR Bass OR Biobloc OR Bioblock OR Bionator OR Bimler OR Crossbow OR Eureka OR Forsus OR Frankel OR Fraenkel OR Fränkel OR Harvold OR Herbst OR "Jasper Jumper" OR MARA OR "Mandibular Advancer" OR "Mandibular Anterior Repositioning" OR Monoblock OR Monobloc OR Sabbagh OR Stockli OR Stöckli OR Stoeckli OR Teuscher OR "Twin Block" OR "Twin force bite corrector") AND (TMJ OR condyle OR temporomandibular OR "mandibular joint" OR "glenoid fossa") AND ("computed tomography" OR "computer tomography" OR "cone beam" OR tomography OR CBCT OR 3D OR "mangetic resonance" OR MRI OR threedimension*) | Humans | 140 |
| Embase | Same as MEDLINE | Humans | 28 |
| Web of Knowledge | Same as MEDLINE | DENTISTRY ORAL SURGERY MEDICINE | 52 |
| Scopus | ( TITLE-ABS-KEY(orthodon* OR malocclusion OR orthopedics OR "Class II" OR prognath* OR retrognath*) AND TITLE-ABS-KEY("functional appliance" OR "functional treatment" OR Activator OR Andresen OR Bass OR Biobloc OR Bioblock OR Bionator OR Bimler OR Crossbow OR Eureka OR Forsus OR Frankel OR Fraenkel OR Fränkel OR Harvold OR Herbst OR "Jasper Jumper" OR MARA OR "Mandibular Advancer" OR "Mandibular Anterior Repositioning" OR Monoblock OR Monobloc OR Sabbagh OR Stockli OR Stöckli OR Stoeckli OR Teuscher OR "Twin Block" OR "Twin force bite corrector") AND TITLE-ABS-KEY(TMJ OR condyle OR temporomandibular OR "mandibular joint" OR "glenoid fossa") AND TITLE-ABS-KEY("computed tomography" OR "computer tomography" OR "cone beam" OR tomography OR CBCT OR 3D OR "mangetic resonance" OR MRI OR threedimension*)) | Dentistry | 65 |
| CDSR | Same as MEDLINE | - | 0 |
| DARE | Same as MEDLINE | - | 1 |
| CENTRAL | Same as MEDLINE | - | 12 |
| Virtual Health Library | Same as MEDLINE | - | 20 |

**Appendix 2**. Additional details for this systematic review and changes from the protocol.

**Deviations from protocol**

- The risk of bias of included non-randomized studies was initially planned to be assessed with the Downs and Black tool that is suggested in the Cochrane Handbook. However, the ROBINS-I ("Risk Of Bias In Non-randomized Studies - of Interventions") tool has been developed in the meantime and is suggested by the Cochrane Collaboration for non-randomized studies, so we switched to this improved tool.
- The number needed to treat was planned to be calculated to clinically translate the results of meta-analyses statistically significant relative risks, but no statistically significant relative risks were ultimately found by meta-analyses.
- Possible sources of heterogeneity were planned a priori in the protocol to be sought through mixed-effects subgroup analyses and random-effects meta-regression for meta-analyses of at least five studies. This could ultimately not be assessed, as less than 5 studies were included in any meta-analysis.
- Reporting biases were planned to be assessed for meta-analyses of at least 10 studies using contour-enhanced funnel plots and with the Egger's weighted regression test. This could ultimately not be assessed, as less than 10 studies were included in any meta-analysis.
- The robustness of the results was planned to be checked a priori with sensitivity analyses based on (i) inclusion/exclusion of trials with low risk of bias and (ii) improvement of the GRADE classification. However, all results were based on one, two, or seldom three trials and therefore any trial omissions were not deemed stable.

**Appendix 3**. List of included/excluded studies with reasons.

| Nr | Paper | Status |
|---|---|---|
| *Excluded by title or abstract* | | |
| 1 | Andresen R, Radmer S, Ludtke CW, Kamusella P, Wissgott C, Schober HC. Balloon sacroplasty as a palliative pain treatment in patients with metastasis-induced bone destruction and pathological fractures. Rofo. 2014;186(9):881-6. | Excluded by title |
| 2 | Aponte-Tinao LA, Piuzzi NS, Roitman P, Farfalli GL. A High-grade Sarcoma Arising in a Patient With Recurrent Benign Giant Cell Tumor of the Proximal Tibia While Receiving Treatment With Denosumab. Clin Orthop Relat Res. 2015;473(9):3050-5. | Excluded by title |
| 3 | Arat FE, Arat ZM, Tompson B, Tanju S. Muscular and condylar response to rapid maxillary expansion. Part 3: Magnetic resonance assessment of condyle-disc relationship. Am J Orthod Dentofacial Orthop 2008;133(6):830-6. | Excluded by title |
| 4 | Aydinli U, Ozturk C, Saba D, Ersozlu S. Neglected major vessel injury after anterior spinal surgery: a case report. Spine (Phila Pa 1976). 2004;29(15):E318-20. | Excluded by title |
| 5 | Batista K, Lima T, Palomares N, Carvalho FA, Quintao C, Miguel JAM, et al. Herbst appliance with skeletal anchorage versus dental anchorage in adolescents with Class II malocclusion: study protocol for a randomised controlled trial. Trials. 2017;18(1):564. | Excluded by title |
| 6 | Behnia H, Motamedi MH, Tehranchi A. Use of activator appliances in pediatric patients treated with costochondral grafts for temporomandibular joint ankylosis: analysis of 13 cases. J Oral Maxillofac Surg. 1997;55(12):1408-14; discussion 14-6. | Excluded by title |
| 7 | Bellintani C, Ghiringhelli P, Gerloni V, Gattinara M, Farronato G, Fantini F. Temporomandibular joint involvement in juvenile idiopathic arthritis: Treatment with an orthodontic appliance. Reumatismo. 2005;57(3):201-7. | Excluded by title |
| 8 | Bender CA, Veneman W, Veenland JF, Mathijssen IM, Hop WC, Koudstaal MJ, et al. Orbital aspects following monobloc advancement in syndromic craniosynostosis. J Craniomaxillofac Surg. 2013;41(7):e146-53. | Excluded by title |
| 9 | Bondemark L. Does 2 years' nocturnal treatment with a mandibular advancement splint in adult patients with snoring and OSAS cause a change in the posture of the mandible? Am J Orthod Dentofacial Orthop 1999;116(6):621-8. | Excluded by title |
| 10 | Ceccarelli AA, Rivas NH, Lorenz GI. Recuperación morfofuncional de la ATM postcondilectomía, tratada con ortopedia funcional de los maxilares. Rev Asoc Argent Ortop Funcional Maxilares. 2001;32(1):27-36. | Excluded by title |
| 11 | Chaudhry A, Sidhu MS, Chaudhary G, Grover S, Chaudhry N, Kaushik A. Evaluation of stress changes in the mandible with a fixed functional appliance: a finite element study. Am J Orthod Dentofacial Orthop. 2015;147(2):226-34. | Excluded by title |
| 12 | Chen J, Sorensen KP, Gupta T, Kilts T, Young M, Wadhwa S. Altered functional loading causes differential effects in the subchondral bone and condylar cartilage in the temporomandibular joint from young mice. Osteoarthritis Cartilage. 2009;17(3):354-61. | Excluded by title |
| 13 | Choi BH. Comparison of computed tomography imaging before and after functional treatment of bilateral condylar fractures in adults. Int J Oral Maxillofac Surg. 1996;25(1):30-3. | Excluded by title |
| 14 | Chung J, Park DH, Yoon SH. Monoblock craniofacial internal distraction in a child with Pfeiffer syndrome: a case report. J Korean Med Sci. 2008;23(2):342-6. | Excluded by title |
| 15 | Cossellu GD, Farronato MD, Biagi RMD, Assandri FMD, Farronato GMD. Idiopathic hypoplasia of the masseter muscle: A case report. Cranio. 2017;35(3):192-6. | Excluded by title |
| 16 | Costa H, Zenha H, Sequeira H, Coelho G, Gomes N, Pinto C, et al. Microsurgical reconstruction of the maxilla: Algorithm and concepts. J Plast Reconstr Aesthet Surg. 2015;68(5):e89-e104. | Excluded by title |
| 17 | De Moraes MEL, Hollender LG, Chen CSK, Moraes LC, Balducci I. Evaluating craniofacial asymmetry with digital cephalometric images and cone-beam computed tomography. Am J Orthod Dentofacial Orthop 2011;139(6):e523-e31. | Excluded by title |
| 18 | Dutra EH, O'Brien MH, Lima A, Kalajzic Z, Tadinada A, Nanda R, et al. Cellular and matrix response of the mandibular condylar cartilage to botulinum toxin. PLoS ONE. 2016;11(10). | Excluded by title |
| 19 | El-Bialy T, Alhadlaq A, Felemban N, Yeung J, Ebrahim A, Hassan AH. The effect of light-emitting diode and laser on mandibular growth in rats. Angle Orthodontist. 2015;85(2):233-8. | Excluded by title |
| 20 | Erbas B, Kocadereli I. Upper airway changes after Xbow appliance therapy evaluated with cone beam computed tomography. Angle Orthod. 2014;84(4):693-700. | Excluded by title |
| 21 | Eto LF, Corrêa FM, Corrêa PH. Correção da pseudo Classe III com aparelho ortopédico de Bimler. Ortho Sci, Orthod sci pract. 2013;6(24):502-6. | Excluded by title |
| 22 | Fagerstrom T, Hedlund R. Cotrel Dubousset instrumentation in occipito-cervico-thoracic fusion. Eur Spine J. 2002;11(4):364-74. | Excluded by title |
| 23 | Falcini F, Melchiorre D, Cappelli S, Carnesecchi G, Biondi K, Bosco M, et al. Temporomandibular joints (TMJ) involvement in juvenile idiopathic arthritis (JIA): Longitudinal evaluation after orthopaedic treatment. Annals of the Rheumatic Disease. 2013;71. | Excluded by title |
| 24 | Falcini F, Melchiorre D, Carnesecchi G, Bertini F, Biondi K, Bosco M, et al. Orthopaedic treatment of temporomandibular joint (TMJ) damage in adolescents with juvenile idiopathic arthritis (JIA): Longitudinal evaluation. Arthritis and Rheumatism. 2012;64:S855. | Excluded by title |
| 25 | Farronato G, Bellintani C, Garagiola U, Cressoni P, Sarzi Puttini P, Atzeni F, et al. Three-dimensional morphological condylar and mandibular changes in a patient with juvenile idiopathic arthritis: interdisciplinary treatment. Reumatismo. 2014;66(3):254-7. | Excluded by title |
| 26 | Fujita T, Ohtani J, Shirakura M, Hayashi H, Kawata T, Kaku M, et al. Changes in cortical bone mineralization in the mouse mandible with regenerated condyle. European Journal of Oral Sciences. 2011;119(2):136-40. | Excluded by title |
| 27 | Gao YZ, Xing S, Gao K, Shi XG, Zhang JY, Yu ZZ, et al. [Anterior retropharyngeal debridement combined with posterior atlantoaxial fusion for atlantoaxial Tuberculosis]. Zhonghua Yi Xue Za Zhi. 2016;96(19):1495-9. | Excluded by title |
| 28 | Garagiola U, Cressoni P, Del Rosso E, Farronato G. Digital titanium-Herbst appliance and psoriatic arthritis: Mandibular and condylar growth effects. Journal of the European Academy of Dermatology and Venereology. 2013;27:55. | Excluded by title |
| 29 | Ghogawala Z, Mansfield FL, Borges LF. Spinal radiation before surgical decompression adversely affects outcomes of surgery for symptomatic metastatic spinal cord compression. Spine (Phila Pa 1976). 2001;26(7):818-24. | Excluded by title |
| 30 | González MFO, Pedersen TK, Dalstra M, Herlin T, Verna C. 3D evaluation of mandibular skeletal changes in juvenile arthritis patients treated with a distraction splint: A retrospective follow-up. Angle Orthodontist. 2016;86(5):846-53. | Excluded by title |
| 31 | Grosfeld O. The orthodontist in the team-treatment for children with rheumatoid arthritis. Eur J Orthod. 1989;11(2):120-4. | Excluded by title |
| 32 | Guo X, Peng J, Wang Y, Wang A, Zhang X, Yuan M, et al. NELL1 promotes bone regeneration in polyethylene particle-induced osteolysis. Tissue Eng Part A. 2012;18(13-14):1344-51. | Excluded by title |
| 33 | Guven O, Keskin A. Remodelling following condylar fractures in children. Journal of Cranio-Maxillofacial Surgery. 2001;29(4):232-7. | Excluded by title |
| 34 | Ha MH, Kim YI, Park SB, Kim SS, Son WS. Cone-beam computed tomographic evaluation of the condylar remodeling occurring after mandibular set-back by bilateral sagittal split ramus osteotomy and rigid fixation. Korean Journal of Orthodontics. 2013;43(6):263-70. | Excluded by title |

| | | |
|---|---|---|
| 35 | Hlawitschka M, Eckelt U. Assessment of patients treated for intracapsular fractures of the mandibular condyle by closed techniques. J Oral Maxillofac Surg. 2002;60(7):784-91; discussion 92. | Excluded by title |
| 36 | Jacquet W, Nyssen E, Bottenberg P, de Groen P, Vande Vannet B. Novel information theory based method for superimposition of lateral head radiographs and cone beam computed tomography images. Dentomaxillofac Radiol. 2010;39(4):191-8. | Excluded by title |
| 37 | Jing D, Yan Z, Cai J, Tong S, Li X, Guo Z, et al. Low-1 level mechanical vibration improves bone microstructure, tissue mechanical properties and porous titanium implant osseointegration by promoting anabolic response in type 1 diabetic rabbits. Bone. 2018;106:11-21. | Excluded by title |
| 38 | Jun W, Yi-Jun K, Fei C. Application of a crescent-shaped PMMA strut in anterior spinal reconstruction of thoracolumbar and lumbar burst fractures: a new technique. Turk Neurosurg. 2012;22(2):161-6. | Excluded by title |
| 39 | Kahl B, Fischbach R, Gerlach KL. Temporomandibular joint morphology in children after treatment of condylar fractures with functional appliance therapy: a follow-up study us computed tomography. Dentomaxillofac Radiol. 1995;24(1):37-45. | Excluded by title |
| 40 | Kahl-Nieke B, Fischbach R, Gerlach KL. CT analysis of temporomandibular joint state in children 5 years after functional treatment of condylar fractures. Int J Oral Maxillofac Surg. 1994;23(6 Pt 1):332-7. | Excluded by title |
| 41 | Kahl-Nieke B, Fischbach R. [A critical evaluation of the functional treatment of mandibular neck fractures in children. The results of a spiral computed tomographic follow-up]. Fortschr Kieferorthop. 1995;56(3):157-64. | Excluded by title |
| 42 | Kahl-Nieke B, Fischbach R. Condylar restoration after early TMJ fractures and functional appliance therapy. Part II: Muscle evaluation. J Orofac Orthop. 1999;60(1):24-38. | Excluded by title |
| 43 | Kahl-Nieke B, Fischbach R. Effect of early orthopedic intervention on hemifacial microsomia patients: an approach to a cooperative evaluation of treatment results. Am J Orthod Dentofacial Orthop. 1998;114(5):538-50. | Excluded by title |
| 44 | Karkabi S, Reis ND, Linn S, Edelson G, Tzehoval E, Zakut V, et al. Pyknodysostosis: imaging and laboratory observations. Calcif Tissue Int. 1993;53(3):170-3. | Excluded by title |
| 45 | Katsumata A, Nojiri M, Fujishita M, Ariji Y, Ariji E, Langlais RP. Condylar head remodeling following mandibular setback osteotomy for prognathism: A comparative study of different imaging modalities. Oral Surgery Oral Medicine Oral Pathology Oral Radiology and Endodontics. 2006;101(4):505-14. | Excluded by title |
| 46 | Kerschbaumer F, Kandziora F, Klein C, Mittlmeier T, Starker M. Transoral decompression, anterior plate fixation, and posterior wire fusion for irreducible atlantoaxial kyphosis in rheumatoid arthritis. Spine (Phila Pa 1976). 2000;25(20):2708-15. | Excluded by title |
| 47 | Khanum A, Kumar S, Mathew S, Shivamurty PG, Pattabiraman V, Naidu M. Stress and displacement pattern in the maxilla and the mandible with reverse twin block: A finite element analysis. Journal of Clinical and Diagnostic Research. 2018;12(7):ZC24-ZC30. | Excluded by title |
| 48 | Kitai N, Kreiborg S, Bakke M, Paulsen HU, Moller E, Darvann TA, et al. Three-dimensional magnetic resonance image of the mandible and masticatory muscles in a case of juvenile chronic arthritis treated with the Herbst appliance. Angle Orthod. 2002;72(1):81-7. | Excluded by title |
| 49 | Kitai N, Kreiborg S, Murakami S, Bakke M, Moller E, Darvann TA, et al. A three-dimensional method of visualizing the temporomandibular joint based on magnetic resonance imaging in a case of juvenile chronic arthritis. Int J Paediatr Dent. 2002;12(2):109-15. | Excluded by title |
| 50 | Kovalko I, Stoustrup P, Benseler S, Twilt M. Update on temporomandibular joint arthritis in juvenile idiopathic arthritis. Journal of Rheumatology. 2018;45(7):1030. | Excluded by title |
| 51 | Lago JCF. Atlas de ortopedia funcional dos maxilares. 1987:181-. | Excluded by title |
| 52 | Li FC, Chen QX, Liu YS, Xu K, Chen WS, Wu QH. [Diagnosis and treatment of odontoid fracture combined with lower cervical spinal injury]. Zhonghua Wai Ke Za Zhi. 2006;44(20):1395-8. | Excluded by title |
| 53 | Li FC, Chen QX, Liu YS, Xu K, Chen WS, Wu QH. [Diagnosis and treatment of odontoid fracture combined with lower cervical spinal injury]. Zhonghua Wai Ke Za Zhi. 2006;44(20):1395-8. | Excluded by title |
| 54 | Li T, Xiao R, Guan Q, Wang M, Dong C. [Improved method of treating thoracolumbar burst fracture by posterior pedicle fixation and grafting through injured vertebrae]. Zhongguo Xiu Fu Chong Jian Wai Ke Za Zhi. 2012;26(5):546-9. | Excluded by title |
| 55 | Liu H, Shi R, Gong Q. [Anterior single segmental decompression and fusion to treat lumbar burst fracture]. Zhongguo Xiu Fu Chong Jian Wai Ke Za Zhi. 2007;21(10):1080-3. | Excluded by title |
| 56 | Lorenz GI, Rivas NH, Ceccarelli A. Las laterodesviaciones mandibulares, fuoncionales y esqueletales. Su resolución desde la ortopedia funcional. Rev Asoc Argent Ortop Funcional Maxilares. 1999;30(1):27-48. | Excluded by title |
| 57 | Lu CL, Song YM, Liu H, Liu LM, Gong Q, Li T, et al. [Application of n-HA/PA66 composite artificial vertebral body in anterior reconstruction of lower cervical spine fracture and dislocation]. Zhonghua Wai Ke Za Zhi. 2012;50(4):338-41. | Excluded by title |
| 58 | Meazzini MC, Brusati R, Diner P, Giann E, Lalatta F, Magri AS, et al. The importance of a differential diagnosis between true hemifacial microsomia and pseudo-hemifacial microsomia in the post-surgical long-term prognosis. Journal of Cranio-Maxillofacial Surgery. 2011;39(1):10-6. | Excluded by title |
| 59 | Meves R, Avanzi O. Correlation between neurological deficit and spinal canal compromise in 198 patients with thoracolumbar and lumbar fractures. Spine (Phila Pa 1976). 2005;30(7):787-91. | Excluded by title |
| 60 | Meyer-Marcotty P, Kochel J, Richter U, Richter F, Stellzig-Eisenhauer A. Reaction of facial soft tissues to treatment with a Herbst appliance. J Orofac Orthop. 2012;73(2):116-25. | Excluded by title |
| 61 | Morris DO, Illing HM, Lee RT. A prospective evaluation of Bass, Bionator and Twin Block appliances. Part II--The soft tissues. Eur J Orthod. 1998;20(6):663-84. | Excluded by title |
| 62 | Mun SH, Won HY, Hernandez P, Aguila HL, Lee SK. Deletion of CD74, a putative MIF receptor, in mice enhances osteoclastogenesis and decreases bone mass. J Bone Miner Res. 2013;28(4):948-59. | Excluded by title |
| 63 | Ono Y, Lin YF, Iijima H, Miwa Z, Ono H. [Two cases of temporomandibular arthrosis of young children]. Nihon Ago Kansetsu Gakkai Zasshi. 1989;1(2):81-93. | Excluded by title |
| 64 | Patil HA, Tekale PD, Kerudi VV, Sharan JS, Lohakpure RA, Mude NN. Assessment of stress changes in dentoalveolar and skeletal structures of the mandible with the miniplate anchored Forsus: A three-dimensional finite element stress analysis study. Apos Trends in Orthodontics. 2017;7(2):87-93. | Excluded by title |
| 65 | Portelli M, Matarese G, Militi A, Logiudice G, Nucera R, Lucchese A. Temporomandibular joint involvement in a cohort of patients with Juvenile Idiopatic Arthritis and evaluation of the effect induced by functional orthodontic appliance: clinical and radiographic investigation. Eur J Paediatr Dent. 2014;15(1):63-6. | Excluded by title |
| 66 | Roitman PD, Jauk F, Farfalli GL, Albergo JI, Aponte-Tinao LA. Denosumab-treated giant cell tumor of bone. Its histologic spectrum and potential diagnostic pitfalls. Hum Pathol. 2017;63:89-97. | Excluded by title |
| 67 | Sahm G. [Success and failure in orthodontic treatment following temporomandibular condylar fractures]. Fortschr Kieferorthop. 1988;49(6):557-67. | Excluded by title |

| | | |
|---|---|---|
| 68 | Salamanna F, Borsari V, Brogini S, Torricelli P, Cepollaro S, Cadossi M, et al. A Human 3D In Vitro Model to Assess the Relationship Between Osteoporosis and Dissemination to Bone of Breast Cancer Tumor Cells. J Cell Physiol. 2017;232(7):1826-34. | Excluded by title |
| 69 | Salgueiro DG, Rodrigues VH, Tieghi Neto V, Menezes CC, Goncales ES, Ferreira Junior O. Evaluation of opening pattern and bone neoformation at median palatal suture area in patients submitted to surgically assisted rapid maxillary expansion (SARME) through cone beam computed tomography. J Appl Oral Sci. 2015;23(4):397-404. | Excluded by title |
| 70 | Sasaki K, Motoyoshi M, Horinuki E, Arai Y, Shimizu N. Effect of low-intensity pulsed ultrasound (LIPUS) on mandibular condyle growth in rats analyzed with micro-CT. Journal of Oral Science. 2016;58(3):415-22. | Excluded by title |
| 71 | Shi R, Liu H, Zhao X, Liu X, Gong Q, Li T, et al. Anterior single segmental decompression and fixation for Denis B type thoracolumbar burst fracture with neurological deficiency: thirty-four cases with average twenty-six month follow-up. Spine (Phila Pa 1976). 2011;36(9):E598-605. | Excluded by title |
| 72 | Skiak E, Karakasli A, Harb A, Satoglu IS, Basci O, Havitcioglu H. The effect of laminae lesion on thoraco-lumbar fracture reduction. Orthop Traumatol Surg Res. 2015;101(4):489-94. | Excluded by title |
| 73 | Spada PP, Moro A, Correr GM, Cevidanes L, Moresca R, Gomes RAP. Avaliação tridimensional por meio de mapas coloridos do tratamento da Classe II com o aparelho de Herbst: relato de caso. Ortho Sci, Orthod sci pract. 2013;6(23):338-45. | Excluded by title |
| 74 | Stoustrup P, Kuseler A, Kristensen KD, Herlin T, Pedersen TK. Orthopaedic splint treatment can reduce mandibular asymmetry caused by unilateral temporomandibular involvement in juvenile idiopathic arthritis. Eur J Orthod. 2013;35(2):191-8. | Excluded by title |
| 75 | Tadej G, Engstrom C, Borrman H, Christiansen EL. Mandibular condyle morphology in relation to malocclusions in children. Angle Orthodontist. 1989;59(3):187-94. | Excluded by title |
| 76 | Tahiri Y, Taylor JA. In support of using computer-aided design and modeling for periorbital osteotomies. J Craniofac Surg. 2015;26(1):100-3. | Excluded by title |
| 77 | Tao HR, Yang TL, Chang MS, Li H, Zhang DW, Fan HB, et al. Successful treatment of a patient with congenital kyphoscoliosis associated with tethered cord. J Neurosurg Spine. 2015;22(1):64-9. | Excluded by title |
| 78 | von Schulze Pellengahr C, von Engelhardt LV, Wegener B, Muller PE, Fottner A, Weber P, et al. Does osteoporosis reduce the primary tilting stability of cementless acetabular cups? BMC Musculoskelet Disord. 2015;16:95. | Excluded by title |
| 79 | Voss A, Shin SR, Murakami AM, Cote MP, Achtnich A, Herbst E, et al. Objective quantification of trochlear dysplasia: Assessment of the difference in morphology between control and chronic patellofemoral instability patients. Knee. 2017;24(5):1247-55. | Excluded by title |
| 80 | Wang C, Wang X, Xu XL, Yuan XL, Gou WL, Wang AY, et al. Bone microstructure and regional distribution of osteoblast and osteoclast activity in the osteonecrotic femoral head. PLoS One. 2014;9(5):e96361. | Excluded by title |
| 81 | Wang L, Liu C, Tian JW. [Surgical treatment strategies of odontoid fractures]. Zhonghua Yi Xue Za Zhi. 2013;93(27):2122-5. | Excluded by title |
| 82 | Wang L, Liu L, Song Y, Pei F, Liu H. Cervical tuberculosis associated with cervical pain and neurologic deficit: a case report and literature review. Spine J. 2014;14(5):e13-8. | Excluded by title |
| 83 | Wang S, Ye L, Li M, Zhan H, Ye R, Li Y, et al. Effects of growth hormone and functional appliance on mandibular growth in an adolescent rat model. Angle Orthodontist. 2018;88(5):624-31. | Excluded by title |
| 84 | Wang XW, Yuan W, Chen DY, Chen XS, Zhou XH, Ye XJ, et al. [Surgical strategy for severe cervical spine dislocations]. Zhonghua Wai Ke Za Zhi. 2007;45(6):379-82. | Excluded by title |
| 85 | Yang X, Song Y, Kong Q, Liu H, Liu L, Gong Q, et al. [Clinical feature and preliminary surgical outcome of traumatic lumbar spondylolisthesis]. Zhongguo Xiu Fu Chong Jian Wai Ke Za Zhi. 2013;27(8):965-8. | Excluded by title |
| 86 | Yildirim Y, Keller EE. Remodeling of displaced condylar fractures with functional treatment: High-quality radiographic documentation in three-patient series. Craniomaxillofacial Trauma and Reconstruction. 2014;8(4):334-40. | Excluded by title |
| 87 | Zhang HQ, Wang YX, Guo CF, Liu JY, Wu JH, Chen J, et al. One-stage posterior approach and combined interbody and posterior fusion for thoracolumbar spinal tuberculosis with kyphosis in children. Orthopedics. 2010;33(11):808. | Excluded by title |
| 88 | Zhang Q, Sun X, Yang J, Ding H, LeBrun D, Ding K, et al. ZIP4 silencing improves bone loss in pancreatic cancer. Oncotarget. 2015;6(28):26041-51. | Excluded by title |
| 89 | Aidar LAdA, Abrahão M, Yamashita HK, Dominguez-Rodriguez GC. Avaliação por meio de ressonância magnética das mudanças da posição dos côndilos nas ATMs de adolescentes com retrognatismo mandibular tratados com aparelho de Herbst. Ortodontia. 2004;37(1):34-49. | Excluded by abstract |
| 90 | Amini M, Heravi F, Zandi B, Eslami S, Mohajerzadeh M, Rohani M. The effect of mandibular advancement device on physiologic parameters and volumetric MRI in mild to moderate obstructive sleep apnea-a randomized controlled trial. Sleep Medicine. 2017;40:e14-e5. | Excluded by abstract |
| 91 | Dominguez-Morea GC. Avaliação das articulações temporomandibulares, à imagens de ressonância magnética e axiografia, de adolescentes com maloclusão classe II, div. 1ª tratados em duas fases (Herbst seguido de aparelho ortodôntico fixo pré-ajustado). 2008:218-. | Excluded by abstract |
| 92 | Foucart JM, Pajoni D, Carpentier P, Pharaboz C. [MRI study of temporomandibular joint disk behavior in chilren with hyperpropulsion appliances]. Orthod Fr. 1998;69(1):79-91. | Excluded by abstract |
| 93 | Franco AdA, Cevidanes LHS, Vigorito JW, Yamashita HK, Lederman HM. Influência dos acessórios ortodônticos na aquisiçäo da imagem por ressonância madnética. Ortodontia. 1999;32(2):15-31. | Excluded by abstract |
| 94 | Gao W, Li X, Bai Y. An assessment of late fixed functional treatment and the stability of Forsus appliance effects. Aust Orthod J. 2014;30(1):2-10. | Excluded by abstract |
| 95 | Hansen K. Post-treatment effects of the Herbst appliance. A radiographic, clinical and biometric investigation. Swed Dent J Suppl. 1992;88:1-49. | Excluded by abstract |
| 96 | Kau CH. The herbst appliance and TMJ morphology: Is there an effect?: Does the herbst appliance have any effect on temporomandibular joint morphology? Evidence-Based Dentistry. 2004;5(4):105. | Excluded by abstract |
| 97 | Kecik D, Kocadereli I, Saatci I. Evaluation of the treatment changes of functional posterior crossbite in the mixed dentition. Am J Orthod Dentofacial Orthop. 2007;131(2):202-15. | Excluded by abstract |
| 98 | Kinzinger GS, Roth A, Gulden N, Bucker A, Diedrich PR. Effects of orthodontic treatment with fixed functional orthopaedic appliances on the condyle-fossa relationship in the temporomandibular joint: a magnetic resonance imaging study (Part I). Dentomaxillofac Radiol. 2006;35(5):339-46. | Excluded by abstract |
| 99 | Lee KY, Park JH, Tai K, Chae JM. Treatment with Twin-block appliance followed by fixed appliance therapy in a growing Class II patient. Am J Orthod Dentofacial Orthop. 2016;150(5):847-63. | Excluded by abstract |
| 100 | Maia S, Raveli DB, dos Santos-Pinto A, Raveli TB, Gomez SP. Computed Tomographic evaluation of a young adult treated with the Herbst appliance. Dental Press Journal of Orthodontics. 2010;15(5):130-6. | Excluded by abstract |
| 101 | Pancherz H. Dentofacial orthopedics or orthognathic surgery: Is it a matter of age? Am J Orthod Dentofacial Orthop 2000;117(5):571-4. | Excluded by abstract |

| | | |
|---|---|---|
| 102 | Panigrahi P, Vineeth V. Biomechanical effects of fixed functional appliance on craniofacial structures. Angle Orthod. 2009;79(4):668-75. | Excluded by abstract |
| 103 | Paulsen HU, Karle A. Computer tomographic and radiographic changes in the temporomandibular joints of two young adults with occlusal asymmetry, treated with the Herbst appliance. Eur J Orthod. 2000;22(6):649-56. | Excluded by abstract |
| 104 | Rohida NS, Bhad W. A clinical, MRI, and EMG analysis comparing the efficacy of twin blocks and flat occlusal splints in the management of disc displacements with reduction. World J Orthod. 2010;11(3):236-44. | Excluded by abstract |
| 105 | Schiavoni R, Grenga V. Long-term follow-up of a young adult patient with a Class II malocclusion treated with a Herbst appliance. World J Orthod. 2009;10(3):243-51. | Excluded by abstract |
| 106 | Shi JJ, Zeng XL. [Magnetic resonance imaging in the assessment of the temporomandibular joint structure following mandibular advancement]. Zhonghua Kou Qiang Yi Xue Za Zhi. 2007;42(11):665-8. | Excluded by abstract |
| 107 | Uematsu H, Ichida T, Masumi S, Morimoto Y, Tanaka T, Konoo T, et al. Diagnostic image analyses of activator treated temporomandibular joint in growth and maturing stages. Cranio. 2002;20(4):254-63. | Excluded by abstract |
| 108 | Ulusoy C, Darendeliler N. Effects of Class II activator and Class II activator high-pull headgear combination on the mandible: a 3-dimensional finite element stress analysis study. Am J Orthod Dentofacial Orthop. 2008;133(4):490.e9-15. | Excluded by abstract |
| 109 | Yang C. Temporomandibular joint disc reposition and condylar regeneration with orthodontic treatment. International Journal of Oral and Maxillofacial Surgery. 2017;46:59. | Excluded by abstract |
| 110 | Yin ZG, Zou Y, Xie ZG, Xie LK, Li ZL. [Clinical observation of assisted occlusion therapy for repositioning temporomandibular joint discs]. Shanghai Kou Qiang Yi Xue. 2016;25(6):755-7. | Excluded by abstract |
| 111 | Yu X, Liu JQ, Yuan LJ, Mao LX, Zhu M, Fang B. [MRI analysis of the effect on mandibular retrusion with anterior disc displacement treated by using Herbst appliance]. Zhonghua Kou Qiang Yi Xue Za Zhi. 2017;52(3):171-5. | Excluded by abstract |
| 112 | Zhang XJ, Zhou CL, Bai YX, Yang XJ, Wang BK. [Relationship between condyle movement and disc position in patients with Class II division 1 malocclusion]. Zhonghua Kou Qiang Yi Xue Za Zhi. 2010;45(1):16-9. | Excluded by abstract |
| *Excluded by fulltext with reasons* | | |
| 113 | Chen WW, Sang T, Huang Z, Wu J. [A cone-beam CT investigation on the effect of two-phase treatment and fixed appliance treatment only on temporomandibular joint]. Zhonghua Kou Qiang Yi Xue Za Zhi. 2016;51(7):410-4. | Excluded; missing fulltext |
| 114 | Dominguez Rodriguez GC. Estudo comparativo radiográfico das mudanças da posição do côndilo mandibular na fossa articular, decorrentes do tratamento com bionator em adolescentes com maloclusão de Classe II, Div. 1ª. 1999:152-. | Excluded; missing fulltext |
| 115 | Mai L, Yao Y, Zhang S, Wang D, Zhang Z. [Comparison of temporomandibular joint changes in adolescent Class II devision 1 malocclusion patients with mandibular retrusion treated with Twin-block and Class II elastics]. Zhonghua Kou Qiang Yi Xue Za Zhi. 2014;49(7):394-8. | Excluded; missing fulltext |
| 116 | Maia S, Raveli DB, Dib LS, Landázuri DG, Raveli TB. Análise tomográfica da articulação temporomandibular no tratamento com Herbst em adulto jovem. Ortodontia. 2010;43(1):71-8. | Excluded; missing fulltext |
| 117 | Sah MK, Fei-wu K, Gang Z, You-chao W. The effect of Herbst treatment on amount and direction changes of temporomandibular joint growth: A short-term investigation of cone-beam computed tomography. International Journal of Oral and Maxillofacial Surgery. 2017;46:237. | Excluded; conference proceeding |
| 118 | Torres Rodríguez LC, González Olazábal MV, Pérez García LM, Pérez Fernández AM. Efecto de Bionator de California en los trastornos temporomandibulares. Gac méd espirit. 2014;16(3). | Excluded; non-healthy patients |
| 119 | Al-Kalaly AA, Wong RW, Cheung LK, Purkayastha SK, Schatzle M, Rabie AB. Evaluation of bone thickness around the mental foramen for potential fixation of a bone-borne functional appliance: a computer tomography scan study. Clin Oral Implants Res. 2010;21(11):1288-93. | Excluded; non-relevant |
| 120 | Masi M, Lederman HM, Yamashita HK, Aidar LAD. Temporomandibular joint evaluation with magnetic resonance imaging in children with functional unilateral posterior crossbite, treated with rapid maxillary expansion. Am J Orthod Dentofacial Orthop 2009;136(2):207-17. | Excluded; non-relevant |
| 121 | Al-Saleh MA, Alsufyani N, Flores-Mir C, Nebbe B, Major PW. Changes in temporomandibular joint morphology in class II patients treated with fixed mandibular repositioning and evaluated through 3D imaging: a systematic review. Orthod Craniofac Res. 2015;18(4):185-201. | Excluded; systematic review |
| 122 | Ivorra-Carbonell L, Montiel-Company JM, Almerich-Silla JM, Paredes-Gallardo V, Bellot-Arcís C. Impact of functional mandibular advancement appliances on the temporomandibular joint - A systematic review. Medicina Oral, Patologia Oral y Cirugia Bucal. 2016;21(5):e565-e72. | Excluded; systematic review |
| 123 | Jimenez-Silva A, Carnevali-Arellano R, Venegas-Aguilera M, Tobar-Reyes J, Palomino-Montenegro H. Temporomandibular disorders in growing patients after treatment of class II and III malocclusion with orthopaedic appliances: a systematic review. Acta Odontologica Scandinavica. 2018;76(4):262-73. | Excluded; systematic review |
| 124 | Machado E, Grehs RA, Cunali PA. Imaging from temporomandibular joint during orthodontic treatment: a systematic review. Dental Press J Orthod. 2011 May-June;16(3):54.e1-7. | Excluded; systematic review |
| 125 | Popowich K, Nebbe B, Major PW. Effect of Herbst treatment on temporomandibular joint morphology: a systematic literature review. Am J Orthod Dentofacial Orthop. 2003;123(4):388-94. | Excluded; systematic review |
| 126 | Xinqi H, Xiao C, Jun L. [Meta-analysis of the condylar position changes produced by functional appliances in class Ⅱ malocclusion]. Hua Xi Kou Qiang Yi Xue Za Zhi. 2016 Dec 1;34(6):589-593. | Excluded; systematic review |
| 127 | Maia S, Raveli DB, Santos-Pinto Ad, Raveli TB, Gomez SP. Avaliação tomográfica no tratamento com Herbst em adulto jovem. Dental Press J Orthod. 2010;15(5):130-6. | Excluded; case report/series |
| 128 | Paulsen HU, Karle A, Bakke M, Herskind A. CT-scanning and radiographic analysis of temporomandibular joints and cephalometric analysis in a case of Herbst treatment in late puberty. Eur J Orthod. 1995;17(3):165-75. | Excluded; case report/series |
| 129 | Proff P, Richter EJ, Blens T, Fanghänel J, Hützen D, Kordaß B, et al. A Michigan-type occlusal splint with spring-loaded mandibular protrusion functionality for treatment of anterior disk dislocation with reduction. Annals of Anatomy. 2007;189(4):362-6. | Excluded; case report/series |
| 130 | Shotell MD. CBCT and Cephalometric Analysis of the TMJ Complex after Treatment Using a MARA Appliance. MSc Thesis, Loma Linda University, 2014. | Excluded; case report/series |
| 131 | Birkebaek L, Melsen B, Terp S. A laminagraphic study of the alterations in the temporo-mandibular joint following activator treatment. Eur J Orthod. 1984;6(4):257-66. | Excluded; no CT/MRI |
| 132 | Conti ACdCF, Freitas MRd, Conti PCR. Avaliação da posição condilar e disfunção temporomandibular em pacientes com má oclusão de Classe II submetidos à protrusão mandibular ortopédica. Rev Dent Press Ortod Ortop Facial (Impr). 2008;13(2):49-60. | Excluded; no CT/MRI |
| 133 | Conti ACdCF. Avaliação da posição condilar e disfunção temporomandibular em pacientes com má oclusão de classe II submetidos a protrusão mandibular ortopédica. 2004:130-. | Excluded; no CT/MRI |
| 134 | Guner DD, Ozturk Y, Sayman HB. Evaluation of the effects of functional orthopaedic treatment on temporomandibular joints with single-photon emission computerized tomography. European Journal of Orthodontics. 2003;25(1):9-12. | Excluded; no CT/MRI |

| | | |
|---|---|---|
| 135 | Keeling SD, Garvan CW, King GJ, Wheeler TT, McGorray S. Temporomandibular disorders after early Class II treatment with bionators and headgears: results from a randomized controlled trial. Semin Orthod 1995;1:149–164. | Excluded; no CT/MRI |
| 136 | Lee RT, Kyi CS, Mack GJ. A controlled clinical trial of the effects of the Twin Block and Dynamax appliances on the hard and soft tissues. Eur J Orthod. 2007;29(3):272-82. | Excluded; no CT/MRI |
| 137 | Lippold C, Hoppe G, Moiseenko T, Ehmer U, Danesh G. Analysis of condylar differences in functional unilateral posterior crossbite during early treatment--a randomized clinical study. J Orofac Orthop. 2008;69(4):283-96. | Excluded; no CT/MRI |
| 138 | Nedeljković N, Glišić B, Marković E, Šćepan I, Stamenković Z. Orthodontic treatment of nongrowing patient with Class II Division 2 malocclusion by Herbst appliance. Vojnosanitetski Pregled. 2009;66(10):840-4. | Excluded; no CT/MRI |
| 139 | Santos-Pinto PRd, Martins LP, Santos-Pinto Ad, Gandini Júnior LG, Raveli DB, Santos-Pinto CCMd. Mandibular growth and dentoalveolar development in the treatment of Class II, division 1, malocclusion using Balters Bionator according to the skeletal maturation. Dental Press J Orthod. 2013;18(4):43-52. | Excluded; no CT/MRI |
| 140 | Schutz TC, Dominguez GC, Hallinan MP, Cunha TC, Tufik S. Class II correction improves nocturnal breathing in adolescents. Angle Orthod. 2011;81(2):222-8. | Excluded; no CT/MRI |
| 141 | Serbesis-Tsarudis C, Pancherz H. "Effective" TMJ and chin position changes in Class II treatment. Angle Orthodontist. 2008;78(5):813-8. | Excluded; no CT/MRI |
| 142 | Elkordy SA, Abouelezz AM, Fayed MM, Attia KH, Ishaq RA, Mostafa YA. Three-dimensional effects of the mini-implant-anchored Forsus Fatigue Resistant Device: A randomized controlled trial. Angle Orthod. 2016;86(2):292-305. | Excluded; TMJ not assessed |
| 143 | Iwasaki T, Takemoto Y, Inada E, Sato H, Saitoh I, Kakuno E, et al. Three-dimensional cone-beam computed tomography analysis of enlargement of the pharyngeal airway by the Herbst appliance. Am J Orthod Dentofacial Orthop. 2014;146(6):776-85. | Excluded; TMJ not assessed |
| 144 | Li X, Zhou HL, Lou XT, Hu Z, Shen G. [Effect of functional appliance on upper airway in adolescent patients with skeletal Class II malocclusion]. Shanghai Kou Qiang Yi Xue. 2017;26(2):222-7. | Excluded; TMJ not assessed |
| 145 | Rizk S, Kulbersh VP, Al-Qawasmi R. Changes in the oropharyngeal airway of Class II patients treated with the mandibular anterior repositioning appliance. Angle Orthod. 2016;86(6):955-61. | Excluded; TMJ not assessed |
| 146 | Schwartz JP, Raveli TB, Schwartz-Filho HO, Raveli DB. Changes in alveolar bone support induced by the Herbst appliance: a tomographic evaluation. Dental Press J Orthod. 2016;21(2):95-101. | Excluded; TMJ not assessed |
| 147 | Hansen K, Pancherz H, Petersson A. Long-term effects of the Herbst appliance on the craniomandibular system with special reference to the TMJ. Eur J Orthod. 1990;12(3):244-53. | Excluded; cross-sectional study |
| 148 | Ruf S, Pancherz H. Long-term TMJ effects of Herbst treatment: a clinical and MRI study. Am J Orthod Dentofacial Orthop. 1998;114(5):475-83. | Excluded; cross-sectional study |
| 149 | Watted N, Witt E, Kenn W. The temporomandibular joint and the disc-condyle relationship after functional orthopaedic treatment: a magnetic resonance imaging study. Eur J Orthod. 2001;23(6):683-93. | Excluded; cross-sectional study |
| 150 | Aidar LA, Abrahao M, Yamashita HK, Dominguez GC. Herbst appliance therapy and temporomandibular joint disc position: a prospective longitudinal magnetic resonance imaging study. Am J Orthod Dentofacial Orthop. 2006;129(4):486-96. | Excluded; no untreated Class II control |
| 151 | Aidar LA, Abrahao M, Yamashita HK, Dominguez GC. Morphological changes of condyles and Helkimo clinical dysfunction index in patients treated with Herbst--orthodontic appliance. Braz Dent J. 2013;24(4):313-21. | Excluded; no untreated Class II control |
| 152 | Aidar LA, Dominguez GC, Abrahao M, Yamashita HK, Vigorito JW. Effects of Herbst appliance treatment on temporomandibular joint disc position and morphology: a prospective magnetic resonance imaging study. Am J Orthod Dentofacial Orthop. 2009;136(3):412-24. | Excluded; no untreated Class II control |
| 153 | Aidar LA, Dominguez GC, Yamashita HK, Abrahao M. Changes in temporomandibular joint disc position and form following Herbst and fixed orthodontic treatment. Angle Orthod. 2010;80(5):843-52. | Excluded; no untreated Class II control |
| 154 | Aras A, Ada E, Saracoglu H, Gezer NS, Aras I. Comparison of treatments with the Forsus fatigue resistant device in relation to skeletal maturity: a cephalometric and magnetic resonance imaging study. Am J Orthod Dentofacial Orthop. 2011;140(5):616-25. | Excluded; no untreated Class II control |
| 155 | Atresh A, Cevidanes LHS, Yatabe M, Muniz L, Nguyen T, Larson B, et al. Three-dimensional treatment outcomes in Class II patients with different vertical facial patterns treated with the Herbst appliance. Am J Orthod Dentofacial Orthop 2018;154(2):238-48.e1. | Excluded; no untreated Class II control |
| 156 | Cacho A, Ono T, Kuboki T, Martin C. Changes in joint space dimension after the correction of Class II division 1 malocclusion. Eur J Orthod. 2015;37(5):467-73. | Excluded; no untreated Class II control |
| 157 | Hamilton SD, Sinclair PM, Hamilton RH. A cephalometric, tomographic, and dental cast evaluation of Frankel therapy. Am J Orthod Dentofacial Orthop. 1987;92(5):427-36. | Excluded; no untreated Class II control |
| 158 | Katsavrias EG, Voudouris JC. The treatment effect of mandibular protrusive appliances on the glenoid fossa for Class II correction. Angle Orthod. 2004;74(1):79-85. | Excluded; no untreated Class II control |
| 159 | Katsavrias EG. The effect of mandibular protrusive (activator) appliances on articular eminence morphology. Angle Orthod. 2003;73(6):647-53. | Excluded; no untreated Class II control |
| 160 | Kinzinger G, Gulden N, Roth A, Diedrich P. Disc-condyle Relationships during Class II Treatment with the Functional Mandibular Advancer (FMA). J Orofac Orthop. 2006;67(5):356-75. | Excluded; no untreated Class II control |
| 161 | Kinzinger G, Kober C, Diedrich P. Topography and morphology of the mandibular condyle during fixed functional orthopedic treatment - a magnetic resonance Imaging study. Journal of Orofacial Orthopedics-Fortschritte Der Kieferorthopadie. 2007;68(2):124-47. | Excluded; no untreated Class II control |
| 162 | Kinzinger GSM, Hourfar J, Kober C, Lisson JA. Mandibular fossa morphology during therapy with a fixed functional orthodontic appliance: A magnetic resonance imaging study. Journal of Orofacial Orthopedics. 2018;79(2):116-32. | Excluded; no untreated Class II control |
| 163 | LeCornu M, Cevidanes LH, Zhu H, Wu CD, Larson B, Nguyen T. Three-dimensional treatment outcomes in Class II patients treated with the Herbst appliance: a pilot study. Am J Orthod Dentofacial Orthop. 2013;144(6):818-30. | Excluded; no untreated Class II control |
| 164 | Lercornu M. Three dimensional treatment outcomes in class II patients treated using Herbst: a pilot study. MSc Thesis, University of North Carolina, 2013. | Excluded; no untreated Class II control |

| | | |
|---|---|---|
| 165 | Liu B, Wang Y, Song F, Liu M, Duan Y, Zhou L. [Cone-beam CT evaluation of the changes in the temporomandibular joint of patients with class II division 1 subdivision malocclusion before and after twin-block treatment]. Hua Xi Kou Qiang Yi Xue Za Zhi. 2013;31(6):610-4. | Excluded; no untreated Class II control |
| 166 | Ma X, Fang B, Dai Q, Xia Y, Mao L, Jiang L. Temporomandibular joint changes after activator appliance therapy: a prospective magnetic resonance imaging study. J Craniofac Surg. 2013;24(4):1184-9. | Excluded; no untreated Class II control |
| 167 | Ma Z, Xie Q, Yang C, Zhang S, Shen Y, Cai X. Changes in the temporomandibular joint space after functional treatment of disk displacement with reduction. J Craniofac Surg. 2015;26(2):e78-81. | Excluded; no untreated Class II control |
| 168 | Pancherz H, Bjerklin K. The Herbst appliance 32 years after treatment. J Clin Orthod. 2015;49(7):442-51. | Excluded; no untreated Class II control |
| 169 | Pancherz H, Ruf S, Thomalske-Faubert C. Mandibular articular disk position changes during Herbst treatment: a prospective longitudinal MRI study. Am J Orthod Dentofacial Orthop. 1999;116(2):207-14. | Excluded; no untreated Class II control |
| 170 | Pancherz H, Sale H, Bjerklin K. Signs and symptoms of TMJ disorders in adults after adolescent Herbst therapy: a 6-year and 32-year radiographic and clinical follow-up study. Angle Orthod. 2015;85(5):735-42. | Excluded; no untreated Class II control |
| 171 | Pinto PRdS. Avaliação do crescimento mandibular e desenvolvimento dentário em crianças com má oclusão de classe II, divisão 1, naturais e induzidos pelo tratamento ortopédico com o bionator de balters. 2009:134-. | Excluded; no untreated Class II control |
| 172 | Ruf S, Pancherz H. Does bite-jumping damage the TMJ? A prospective longitudinal clinical and MRI study of Herbst patients. Angle Orthod. 2000;70(3):183-99. | Excluded; no untreated Class II control |
| 173 | Ruf S, Pancherz H. Long-term effects of Herbst treatment: A clinical and MRI study. Am J Orthod Dentofacial Orthop 1998;114(5):475-83. | Excluded; no untreated Class II control |
| 174 | Ruf S, Pancherz H. Temporomandibular joint growth adaptation in Herbst treatment: a prospective magnetic resonance imaging and cephalometric roentgenographic study. Eur J Orthod. 1998;20(4):375-88. | Excluded; no untreated Class II control |
| 175 | Ruf S, Pancherz H. Temporomandibular joint remodeling in adolescents and young adults during Herbst treatment: A prospective longitudinal magnetic resonance imaging and cephalometric radiographic investigation. Am J Orthod Dentofacial Orthop. 1999;115(6):607-18. | Excluded; no untreated Class II control |
| 176 | Ruf S, Wusten B, Pancherz H. Temporomandibular joint effects of activator treatment: a prospective longitudinal magnetic resonance imaging and clinical study. Angle Orthod. 2002;72(6):527-40. | Excluded; no untreated Class II control |
| 177 | Souki BQ, Vilefort PLC, Oliveira DD, Andrade I, Jr., Ruellas AC, Yatabe MS, et al. Three-dimensional skeletal mandibular changes associated with Herbst appliance treatment. Orthodontics and Craniofacial Research. 2017;20(2):111-8. | Excluded; no untreated Class II control |
| 178 | VanLaecken R, Martin CA, Dischinger T, Razmus T, Ngan P. Treatment effects of the edgewise Herbst appliance: a cephalometric and tomographic investigation. Am J Orthod Dentofacial Orthop. 2006;130(5):582-93. | Excluded; no untreated Class II control |
| 179 | Wadhawan N, Kumar S, Kharbanda OP, Duggal R, Sharma R. Temporomandibular joint adaptations following two-phase therapy: an MRI study. Orthod Craniofac Res. 2008;11(4):235-50. | Excluded; no untreated Class II control |
| 180 | Weiwei C, Ting S, Zhen H, Jun W. [Cone beam computed tomography analysis of the bony structure of the temporomandibular joint during two phase treatment with Herbst appliance]. Hua Xi Kou Qiang Yi Xue Za Zhi. 2016;34(5):498-501. | Excluded; no untreated Class II control |
| 181 | Yildirim E, Karacay S, Erkan M. Condylar response to functional therapy with Twin-Block as shown by cone-beam computed tomography. Angle Orthod. 2014;84(6):1018-25. | Excluded; no untreated Class II control |
| Included | | |
| 182 | Arat ZM, Gokalp H, Erdem D, Erden I. Changes in the TMJ disc-condyle-fossa relationship following functional treatment of skeletal Class II Division 1 malocclusion: a magnetic resonance imaging study. Am J Orthod Dentofacial Orthop. 2001;119(3):316-9. | Included |
| 183 | Arici S, Akan H, Yakubov K, Arici N. Effects of fixed functional appliance treatment on the temporomandibular joint. Am J Orthod Dentofacial Orthop. 2008;133(6):809-14. | Included |
| 184 | Cevidanes LH, Franco AA, Gerig G, Proffit WR, Slice DE, Enlow DH, et al. Assessment of mandibular growth and response to orthopedic treatment with 3-dimensional magnetic resonance images. Am J Orthod Dentofacial Orthop. 2005;128(1):16-26. | Included |
| 185 | Cevidanes LH, Franco AA, Gerig G, Proffit WR, Slice DE, Enlow DH, et al. Comparison of relative mandibular growth vectors with high-resolution 3-dimensional imaging. Am J Orthod Dentofacial Orthop. 2005;128(1):27-34. | Included |
| 186 | Chavan SJ, Bhad WA, Doshi UH. Comparison of temporomandibular joint changes in Twin Block and Bionator appliance therapy: a magnetic resonance imaging study. Prog Orthod. 2014;15:57. | Included |
| 187 | Chintakanon K, Sampson W, Wilkinson T, Townsend G. A prospective study of Twin-block appliance therapy assessed by magnetic resonance imaging. Am J Orthod Dentofacial Orthop. 2000;118(5):494-504. | Included |
| 188 | Chintakanon K. A Prospective Study of Twin Block Appliance Therapy in Children with Class ll Divisionl Malocclusions Assessed by MRl, 3D-Cephalometry and Muscle Testing. Doctoral Dissertation, University of Adelaide, 1999. | Included |
| 189 | Croft RS, Buschang PH, English JD, Meyer R. A cephalometric and tomographic evaluation of Herbst treatment in the mixed dentition. Am J Orthod Dentofacial Orthop. 1999;116(4):435-43. | Included |
| 190 | Elfeky HY, Fayed MS, Alhammadi MS, Soliman SAZ, El Boghdadi DM. Three-dimensional skeletal, dentoalveolar and temporomandibular joint changes produced by Twin Block functional appliance. Journal of Orofacial Orthopedics. 2018;79(4):245-58. | Included |
| 191 | Franco AA, Yamashita HK, Lederman HM, Cevidanes LH, Proffit WR, Vigorito JW. Frankel appliance therapy and the temporomandibular disc: a prospective magnetic resonance imaging study. Am J Orthod Dentofacial Orthop. 2002;121(5):447-57. | Included |

| | | |
|---|---|---|
| 192 | Franco AdA. Estudo comparativo das ATMs e das estruturas dento-esqueléticas da face, pela ressonância magnética e cefalometria radiográfica em pacientes com má oclusão de classe II divisão 1, tratados com regulador de função d Frãnkel-2 e em indivíduos com oclusão normal. 2004:[184]-[]. | Included |

_

**Appendix 4**. List of included studies with their analyzed outcomes.

| Nr | Study | Outcome | MD | 95% CI | P | $SD_{CTR}$ | Clinically relevant |
|---|---|---|---|---|---|---|---|
| 1 | Chintakanon 1999; 2000 | Anterior angle (FH as reference) | 3.6 | -1.84, 9.04 | 0.2 | 11.1 | - |
| 2 | Arat 2001 | Anterior angle (PC as reference) | 3.08 | -2.04, 8.20 | 0.24 | 4.3 | - |
| 3 | Chintakanon 1999; 2000 | Anterior angle (PC as reference) | -1.5 | -5.77, 2.77 | 0.49 | 10.1 | - |
| 4 | Arat 2001 | Anterior joint space | -0.65 | -0.87, -0.43 | <0.001 | 0.2 | Yes |
| 5 | Elfeky 2018* | Anterior joint space | -0.84 | -1.14, -0.54 | <0.001 | 0.48 | Yes |
| 6 | Arici 2008 | Anterior joint space volume | 30 | 17.20, 42.81 | <0.001 | 45 | No |
| 7 | Arici 2008 | Anterior-posterior joint space volume | 46 | 31.05, 60.95 | <0.001 | 67 | No |
| 8 | Chintakanon 1999; 2000 | Condylar axial angle | 4.7 | -0.97, 10.37 | 0.1 | 9.2 | - |
| 9 | Elfeky 2018* | Condylar coronary AP length | 0.53 | 0.21, 0.85 | 0.001 | 1.16 | No |
| 10 | Elfeky 2018* | Condylar coronary height | 1.11 | 0.64, 1.58 | <0.001 | 1.36 | No |
| 11 | Chintakanon 1999; 2000 | Condylar coronary width | 0.5 | -0.34, 1.34 | 0.24 | 2.1 | - |
| 12 | Elfeky 2018* | Condylar coronary width | 1.6 | 1.31, 1.89 | <0.001 | 1.93 | No |
| 13 | Chintakanon 1999; 2000 | Condylar head shape: convex | RR: 1.53 | 1.08, 2.18 | 0.02 | | No |
| 14 | Chintakanon 1999; 2000 | Condylar head shape: flat | RR: 0.12 | 0.01, 2.13 | 0.15 | | - |
| 15 | Chintakanon 1999; 2000 | Condylar head shape: round | RR: 0.28 | 0.03, 2.26 | 0.23 | | - |
| 16 | Chintakanon 1999; 2000 | Condylar position: concentric | RR: 0.66 | 0.18, 2.41 | 0.53 | | - |
| 17 | Chintakanon 1999; 2000 | Condylar position: concentric at end but not at start | RR: 0.38 | 0.05, 2.96 | 0.35 | | - |
| 18 | Elfeky 2018* | Condyle sagittal displacement | 1.3 | 0.92, 1.68 | <0.001 | 2.43 | No |
| 19 | Franco 2002* | Condyle to articular tubercle | 1.4 | 1.30, 1.50 | <0.001 | 0.28 | Yes |
| 20 | Franco 2002* | Condyle to eminence convexity change | 1.19 | 0.84, 1.54 | <0.001 | 0.37 | Yes |
| 21 | Elfeky 2018* | Condyle transverse displacement | -0.07 | -0.55, 0.41 | 0.78 | 3.04 | - |
| 22 | Elfeky 2018* | Condyle vertical displacement | -0.59 | -1.07, -0.11 | 0.02 | 0.86 | No |
| 23 | Arici 2008 | Condyle volume | 11 | 1.16, 20.84 | 0.03 | 57 | No |
| 24 | Franco 2002 | Disc displacement (closed mouth) | RR: 0.20 | 0.01, 3.99 | 0.29 | | - |
| 25 | Franco 2002 | Disc displacement (open mouth) | RR: 0.20 | 0.01, 3.99 | 0.29 | | - |
| 26 | Chintakanon 1999; 2000 | Disc position: centric | RR: 0.66 | 0.18, 2.41 | 0.53 | | - |
| 27 | Chintakanon 1999; 2000 | Disc position: lateral | RR: 1.24 | 0.60, 2.56 | 0.55 | | - |
| 28 | Chintakanon 1999; 2000 | Disc position: medial | RR: 0.97 | 0.43, 2.16 | 0.94 | | - |
| 29 | Franco 2002 | Disc shape: nonbiconcave | RR: 0.09 | 0.01, 1.57 | 0.1 | | - |
| 30 | Chintakanon 1999; 2000 | Eminence angle (FH as reference) | -0.7 | -7.58, 6.18 | 0.84 | 10.7 | - |
| 31 | Chintakanon 1999; 2000 | Eminence angle (PC as reference) | 4.7 | -2.83, 12.23 | 0.22 | 13.6 | - |
| 32 | Chintakanon 1999; 2000 | FH-PC angle | 4.5 | 1.28, 7.72 | 0.006 | 9.3 | No |
| 33 | Croft 1999 | Glenoid fossa sagittal displacement | -0.1 | -0.64, 0.44 | 0.72 | 1.4 | - |
| 34 | Elfeky 2018* | Glenoid fossa sagittal displacement | -0.55 | -1.17, 0.07 | 0.08 | 2.39 | No |
| 35 | Croft 1999 | Glenoid fossa sagittal/vertical displacement | 0.7 | 0.19, 1.21 | 0.007 | 1.2 | No |
| 36 | Elfeky 2018* | Glenoid fossa transverse displacement | 0.41 | -0.51, 1.33 | 0.38 | 1.68 | - |
| 37 | Croft 1999 | Glenoid fossa vertical displacement | -0.2 | -0.85, 0.45 | 0.55 | 1.3 | - |
| 38 | Elfeky 2018* | Glenoid fossa vertical displacement | -0.45 | -0.80, -0.10 | 0.01 | 0.47 | No |
| 39 | Arici 2008 | Glenoid fossa volume | 8 | -1.48, 17.48 | 0.1 | 90 | - |
| 40 | Arat 2001 | Medial angle (PC as reference) | 1.25 | -2.86, 5.36 | 0.55 | 3.86 | - |
| 41 | Elfeky 2018* | Medial joint space | -0.67 | -0.89, -0.45 | <0.001 | 1 | No |
| 42 | Chavan 2014 | Posterior angle (Bionator; PC as reference) | -13.8 | -20.64, -6.96 | <0.001 | 9.9 | Yes |
| 43 | Chintakanon 1999; 2000 | Posterior angle (FH as reference) | 3.2 | -2.38, 8.78 | 0.26 | 11.6 | - |
| 44 | Arat 2001 | Posterior angle (PC as reference) | -2.21 | -5.13, 0.71 | 0.14 | 2.58 | - |
| 45 | Chintakanon 1999; 2000 | Posterior angle (PC as reference) | -4 | -12.08, 4.08 | 0.33 | 12.9 | - |
| 46 | Chavan 2014 | Posterior angle (Twin Block/Bionator; PC as reference) | -15.3 | -19.70, -10.90 | <0.001 | 9.9 | Yes |
| 47 | Chavan 2014 | Posterior angle (Twin Block; PC as reference) | -16.8 | -22.47, -11.13 | <0.001 | 9.9 | Yes |
| 48 | Arat 2001 | Posterior joint space | 1.01 | 0.83, 1.19 | <0.001 | 0.24 | Yes |
| 49 | Elfeky 2018* | Posterior joint space | 1.12 | 0.73, 1.51 | <0.001 | 0.67 | No |
| 50 | Arici 2008 | Posterior joint space volume | -16 | -24.04, -7.96 | <0.001 | 43 | No |
| 51 | Franco 2002* | Sagittal concentricity | -10.73 | -13.03, -8.44 | <0.001 | 3.72 | Yes |
| 52 | Chavan 2014 | Sagittal concentricity (Bionator) | 11.55 | 7.15, 15.95 | <0.001 | 5.1 | Yes |
| 53 | Chavan 2014 | Sagittal concentricity (Twin Block) | 15.18 | 10.28, 20.08 | <0.001 | 5.1 | Yes |
| 54 | Chavan 2014 | Sagittal concentricity (Twin Block/Bionator) | 13.37 | 10.06, 16.68 | <0.001 | 5.1 | Yes |
| 55 | Arat 2001 | Superior joint space | 0.64 | 0.35, 0.93 | <0.001 | 0.31 | Yes |
| 56 | Elfeky 2018* | Superior joint space | 0.89 | 0.45, 1.33 | <0.001 | 0.76 | No |

*separate measurements for right and left TMJs were given—the left one was taken in random.

**Appendix 5**. Detailed assessment of the quality from performed meta-analyses according to the GRADE approach used to construct Table 4.

| Outcome | RoB | Indirectness | Inconsistency | Imprecision | Publication bias | Large effect | Dose response | Confounding |
|---|---|---|---|---|---|---|---|---|
| Anterior joint space | **Serious** | **Ok** | **No** | **Ok** | **NA** | **Yes†** | **NA** | **Still existing** |
| Posterior joint space | **Serious** | **Ok** | **No** | **Ok** | **NA** | **Yes†** | **NA** | **Still existing** |
| Superior joint space | **Serious** | **Ok** | **No** | **Ok** | **NA** | **Yes†** | **NA** | **Still existing** |
| Anterior angle | **Serious** | **Ok** | **No** | **Serious** | **NA** | **No** | **NA** | **Still existing** |
| Posterior angle | **Serious** | **Ok** | **Mild*** | **Serious** | **NA** | **Yes†** | **NA** | **Still existing** |
| Condylar coronary width | **Serious** | **Ok** | **Mild*** | **Serious** | **NA** | **No** | **NA** | **Still existing** |
| GleFo sagittal displacement | **Serious** | **Ok** | **No** | **Ok** | **NA** | **No** | **NA** | **Still existing** |
| GleFo vertical displacement | **Serious** | **Ok** | **No** | **Ok** | **NA** | **No** | **NA** | **Still existing** |
| Sagittal concentricity index | **Serious** | **Ok** | **Serious** | **Serious** | **NA** | **No** | **NA** | **Still existing** |

* heterogeneity is high, but all studies are on the same side of the forest plot—i.e. heterogeneity affects only the precise quantification of the treatment effects, but not the decision whether treatment is effective or not.

† Large or very large effect magnitude is seen, but quality of evidence is not upgraded due to existing methodological limitations/risk of bias
